# Population and herbarium genomics provide a comprehensive framework for a revision of *Microcoleus* (Cyanobacteria)

**DOI:** 10.1111/jpy.70145

**Published:** 2026-03-18

**Authors:** Svatopluk Skoupý, Aleksandar Stanojković, Jeffrey R. Johansen, Dale A. Casamatta, Callahan McGovern, Anne D. Jungblut, Jutta Fastner, Petr Dvořák

**Affiliations:** ^1^ Department of Botany, Faculty of Science Palacký University Olomouc Olomouc Czech Republic; ^2^ National Marine Fisheries Research Institute Department of Fisheries Oceanography and Microbial Ecology Gdynia Poland; ^3^ Department of Biology John Carroll University Cleveland Ohio USA; ^4^ Department of Botany, Faculty of Science University of South Bohemia České Budějovice Czech Republic; ^5^ Department of Biology, College of Arts and Sciences University of North Florida Florida USA; ^6^ Department of Science Natural History Museum London UK; ^7^ Section Drinking Water Resources German Environment Agency Berlin Germany

**Keywords:** cyanobacteria, herbaria, *Microcoleus*, population genomics, taxonomy

## Abstract

*Microcoleus* is a cosmopolitan, filamentous cyanobacterium and a key component of biological soil crusts—complex microbial communities essential for primary production in diverse terrestrial environments. Here, we performed a taxonomic revision of several species of *Microcoleus* based on a large population genomic dataset. The dataset was based on a *Microcoleus* speciation continuum characterized by variable levels of gene flow between the species. The putative species ranged from cryptic to distinctly morphologically defined lineages. We identify the type herbarium specimen and obtained a genome for the type species *M. vaginatus* and herein describe 10 novel species of *Microcoleus.* We provide epitypifications for the previously described species *M. vaginatus* and *M. attenuatus*. This research contributes to a more comprehensive understanding of terrestrial cyanobacterial biodiversity and cryptic species in cyanobacteria. It highlights the need for an extensive genomic and phenotypic dataset in the taxonomy of Cyanobacteria.

**Abbreviations**
ANI: average nucleotide identity
API: atmospheric pressure ionization
DIC: differential interference contrast
HPLC: high performance liquid chromatography
HR: homologous recombination
ITS: internal transcribed spacer
LC–MS/MS: liquid chromatography mass spectrometry/mass spectrometry
NCBI: National Center for Biotechnology Information
UPCEL: universal probabilistic concept of evolutionary lineages
WGS: whole‐genome sequencing

## INTRODUCTION

Cryptic diversity coupled with ambiguous species concepts and delimitations represent key issues in current Cyanobacterial taxonomy (Dvořák et al., 2023; Engene et al., 2018; Erwin & Thacker, 2008; Johansen et al., 2017; Skoupý et al., 2024). These factors, together with reliance on morphological features as a main criterion for phenetic species identifications throughout the 19th and 20th centuries, have led to the existence of many cryptic and unrevised species in databases (Dvořák et al., 2018). These factors also pose a problem when describing new taxa. Since most species, historically, were described based on the morphology of a single natural sample or strain, it is difficult to link these species to real populations in nature (Komárek, 2010). Scant, if any, molecular data are available for many Cyanobacterial species in public sequence databases (Komárek et al., 2014), and type material for many species is often very old (up to 200 years; Dvořák et al., 2020) and, in some cases, non‐existent or lost. This situation creates issues when trying to match newly isolated strains to existing species that do not have available molecular data, which has often led to misidentification of species and orphan taxa.

*Microcoleus* is an important genus of fasciculate filamentous cyanobacteria with a cosmopolitan distribution. *Microcoleus vaginatus*, the type species for this genus, inhabits predominantly benthic and subaerophytic habitats and is often a pioneering organism of terrestrial and aquatic environments. *Microcoleus vaginatus* is among the most important microbial primary producers in terrestrial habitats and forms a crucial component of biological soil crusts around the world (Garcia‐Pichel et al., 1996). Despite its crucial role in global ecosystems, the diversity and distribution of *Microcoleus* is still poorly understood. There are currently >3800 articles on Google scholar (as of March 2025) concerning *M. vaginatus*, covering various fields from taxonomy (Skoupý et al., 2024), evolution, and ecology (Nelson et al., 2021) to biotechnology (Lan et al., 2015) and bioremediation (Sabarinathan et al., 2021). Although *M. vaginatus* has usually been considered a single species in the literature, some research has noted the existence of several species within *M. vaginatus* (Boyer et al., 2002; Strunecký et al., 2013).

*Microcoleus* was first erected in 1823 by the French naturalist Jean B. H. J. Desmaziéres, with the type *M. terrestris* described from agricultural soil in France. This species was later synonymized with *M. vaginatus* (Gomont, 1892), which is now considered the type species for *Microcoleus*. *Microcoleus vaginatus* is usually described as mat‐forming, blue green in color, with trichomes 3–7 μm wide, with cells usually two times wider than they are long, with thick distinct sheaths, and with multiple trichomes per sheath (Komárek & Anagnostidis, 2005). Trichomes are usually attenuated toward a calyptrate apical cell. Due to its described cosmopolitan distribution, *M. vaginatus* became a sort of “round‐up” species, commonly described from around the world based on its distinct morphology (e.g., the presence of sheaths and calyptrate apical cells).

Drouet (1962) was perhaps the first author to attempt a large‐scale taxonomic revision of *Microcoleus*. As a proponent of the ecophene theory, Drouet held that the phenotypes of Cyanobacteria are largely shaped by their environments and, accordingly, united a number of morphologically similar genera and species into *Microcoleus* (e.g., Drouet considered *Oscillatoria viridis*, *O. subfusca*, *O. fusca*, *O. autumnalis*, *Phormidium vulgare*, *P. viride*, *P. rupestre*, and many other species as synonyms for *M. vaginatus*). With the advent of molecular methods and genetic sequencing, it became clear that Drouet's view that ecological factors, rather than genetic divergence and evolution, shape the diversity of Cyanobacteria was rather simplistic and did not reflect the extensive species diversity within *Microcoleus*.

The next major effort to resolve the taxonomy of *Microcoleus* was done by Boyer et al. (2002) on a dataset of 31 desert strains of *M. vaginatus* and *M. steenstrupii*. Although Boyer et al. (2002), included both 16S rRNA gene phylogeny and robust morphological analysis and resolved the position of the *Microcoleus* genus among the Oscillatoriales, they did not completely resolve the position of the type species, *M. vaginatus* and noted a further need of revisions. Strunecký et al. (2013) further explored the complicated taxonomy of *Microcoleus*, analyzing 92 strains of *M. vaginatus* and *Phormidium autumnale*. Strunecký et al. (2013) erected a novel family, *Microcoleaceae*, and transferred the *Phormidium* group VII (Komárek & Anagnostidis, 2005) into *Microcoleus*. Although that study was likely the most phylogenetically supported effort for taxonomic revision of *Microcoleus* at the time, Strunecký et al. (2013) did not resolve the taxonomic positions of *M. vaginatus* and phylogenetically closely related *Microcoleus* species and noted the need for further revisions.

In our previous studies, we constructed a large dataset of cultivated *Microcoleus* strains and whole‐genome sequences and showed that species within the *M. vaginatus* group have been diversifying into a speciation continuum (Stanojković et al., 2024). The speciation continuum diversification has been driven by geographic distance, environmental selection, and a significant amount of gene flow by homologous recombination and horizontal gene transfer (Stanojković et al., 2024). In Skoupý et al. (2024), we further explored this continuum. We expanded the genomic dataset and identified 21 putative species of *Microcoleus* in a speciation continuum. We investigated the morphological diversity of 180 strains to evaluate the extent of cryptic species within *Microcoleus*; a whole spectrum from distinctly morphologically defined species to cryptic species was identified. Moreover, we tested the hypothesis that the apical tip shapes had an adaptive function (Garcia‐Pichel et al., 1996; Parret et al., 2023) by analyzing environmental variables. Some of these variables (e.g., soil density, precipitation, and solar radiation) correlated with the shape of the filament apex, suggesting an adaptive function. Thus, these observations suggest that the shape and attenuation of the filaments might serve as an identifying trait for members of the *M. vaginatus* group. Due to the scope of the current manuscript and sampling design, we did not include species of *Microcoleus* lacking calyptrate apical cells, for example, *M. lacustris*, *M. codii*, or *M. subtorulosus*.

In this study, we were able to identify the type material for *Microcoleus vaginatus* sensu stricto, as well as resolve the taxonomy of the *M. vaginatus* group. We matched until‐now putative species to previously established species and have proposed several novel species in cases in which this is not possible using genomics, 16S rRNA gene phylogenetics of cultured cyanobacteria, and herbarium specimens. The goal of this study was to advance the understanding of the biodiversity of terrestrial cyanobacteria and further resolve the taxonomy of the speciation continuum of the important cosmopolitan soil‐crust forming Cyanobacterium *Microcoleus*.

## GLOSSARY

**Calyptra**: thickened cap present in the apices of mature trichomes (Note: an important diagnostic character, but not always present, depending on physiological state and age, for example, phenotypically plastic)

**Constrictions**: invaginations (visible via light or electron microscopy) between adjacent cells

**Filament**: term encompassing both the trichome and sheath, unless sheath‐less (in which case, the trichome without a sheath)

**Granules**: a general term for one of numerous types of cellular inclusions, often employed by bacteria for storage functions; granular placement and forms in cells may be a taxonomically informative feature (e.g., concentrated near cell walls vs interior)

**Hormogonia**: mode of reproduction in which short sections of adjacent cells are liberated from the original trichome via fragmentation or the presence of necridic cells and could be taxonomically informative

**Keritomization**: seemingly vacuolized cells; a net‐like appearance of cyanobacterial protoplast

**Necridic cells**: apoptotically derived cells used to generate hormogonium via cell senescence; not present in all lineages and may be taxonomically informative

**Pointiness:** approximation of the filament apex shape to the pointiness of an ellipse (Skoupý et al., 2024).

**Trichome**: row of connected cells, sans enveloping sheath

## MATERIALS AND METHODS

### Sampling and strain isolation

Cyanobacterial samples were collected from diverse climates and habitats, such as soil crusts, freshwater benthos, moss, and rock surfaces. Sample collection and strain isolation was described in Stanojković et al. (2022). Strains were isolated according to Andersen (2005) and maintained in liquid Zehnder medium (Z‐medium; Staub, 1961) under the following conditions: temperature, 22 ± 1°C; illumination, 20 μmol photons · m^−2^ · s^−1^, and light regime, 12:12 h (light:dark). In total, 495 clonal cultures of *Microcoleus* spp. were obtained (Stanojković et al., 2022). One hundred and sixty selected strains are being maintained in the culture collection at the Department of Botany, Palacký University in Olomouc, Czech Republic. Furthermore, eight herbarium specimens from the herbarium collections of the Natural History Museum (London, United Kingdom; Stanojković et al., 2024; Skoupý et al., 2024) were included (Table 1). DNA extractions and genomic sequencing was previously described in Stanojković et al. (2024). The DNA extraction from herbarium specimen was performed according to ancient DNA extraction protocols (see application of protocols by Kistler, 2012 and Meyer & Kircher, 2010 in Stanojković et al., 2024).

**TABLE 1:** Herbarium specimens of *Microcoleus* spp. held at the Natural History Museum (London, United Kingdom) analyzed in this study.

Species on a specimen	Collected by	Barcode	Accession number
*Microcoleus terrestris*	J.B. Desmazières 1825–1851, France	BM001213989	JAUBOM000000000
*Microcoleus vaginatus*	F.E. Drouet 1938, USA	BM001213995	JAUPTD000000000
*Microcoleus vaginatus*	F.B. Wartmann 1855, Germany	BM001213992	JAUBOL000000000
*Microcoleus vaginatus*	J.A.P. Hepp 1857, Switzerland	BM001150542	JAUBOK000000000
*Phormidium subfuscum*	W.A. Setchell 1892 USA	BM001215392	JAUBOJ000000000
*Phormidium subfuscum*	L.J. von Heufler 1864 Italy	BM001215508	JAUBOI000000000
*Phormidium subfuscum*	V.B. Wittrock 1866 Sweden	BM001215366	JAUBOH000000000
*Phormidium uncinatum*	H.L.D. 1896 USA	BM001215583	JAUBOG000000000

*Note*: **Barcode** refers to the physical specimen held at the Natural History Museum. **Accession number** refers to the whole genome sequence in the NCBI database.

### PCR amplification, DNA extraction, and molecular analysis

The isolation of the genomic DNA and acquisition of the 16S rRNA gene and whole‐genome sequences was described in depth in Stanojković et al. (2022, 2024). A 16S rRNA gene phylogenetic tree was constructed using a dataset of 967 16S rRNA gene sequences. This dataset included sequences from 495 cultivated *Microcoleus* strains, as reported by Stanojković et al. (2022) and eight sequences obtained from herbarium‐derived genomes using the Barrnap tool (https://github.com/tseemann/barrnap), with the remaining sequences retrieved from the National Center for Biotechnology Information (NCBI) database via the BLAST algorithm. Multiple sequence alignment was performed using the MUSCLE algorithm (Edgar, 2004) within the AliView software (Larsson, 2014), and the phylogenetic tree was constructed using IQ‐Tree software v3.0.0 (Wong et al., 2025) with default parameters and bootstraps approximated using ultrafast approximation UFBoot (Hoang et al., 2018; Minh et al., 2013) with TIM2 + I + G model selected by modeltest (Kalyaanamoorthy et al., 2017) in 1000 replicates.

A total of 201 strains were selected for whole‐genome sequencing (WGS; Stanojković et al., 2024). Whole‐genome sequencing was done commercially on the Illumina NovaSeq 6000 platform (Novogene, United Kingdom). Genomes were assembled using SPAdes v3.13.1 (Prjibelski et al., 2020) and annotated using prokka v1.14.5 (Seemann, 2014). The details on our WGS and subsequent genome assembly and annotation were described in Stanojković et al. (2024), including scripts stored at github (https://github.com/dvorikus/Microcoleus‐population‐genomics). The phylogenomic tree was based on Dataset II (Skoupý et al., 2024) and was constructed using Orthofinder v2.3.196 (Emms & Kelly, 2019) and IQ‐TREE v1.6.197 (Nguyen et al., 2015). The best‐fitting model selected by ModelFinder98 (Kalyaanamoorthy et al., 2017) was LG + I + G, and branch supports were computed using ultrafast bootstrapping with 2000 replicates. The phylogenomic analysis was described more in depth in Stanojković et al. (2024). The phylogenomic tree was modified in figtree v1.3.1 (Rambaut, 2010) and inkscape (https://inkscape.org/). We tested several algorithms to elucidate the population structure of *Microcoleus* isolates and to assign them to genetically distinct clusters. The hierarchical Bayesian clustering algorithm implemented in fastBAPS (Tonkin‐Hill et al., 2019) was selected as the most suitable, since it fit the monophyletic clades in the phylogenomic reconstruction (Stanojković et al., 2024). The population genomic clustering was described in depth in Stanojković et al. (2024).

### Morphology assessment

Morphology of 180 selected isolated cyanobacterial strains was studied via light microscopy using a Zeiss AxioImager (objectives EC Plan‐Neofluar 40×/1.3 N.A., oil immersion, differential interference contrast; Plan‐Apochromat 100×/1.4 N.A., oil immersion, differential interference contrast) and documented via high resolution camera (Axio Cam D512 12MPx). Not all strains were viable enough for morphological assessment, but in each clade from our dataset, there were at least three strains available. The following traits were observed for each strain: cell shape and dimensions (cell width and cell length between fully formed cross walls), color, sheath presence and thickness, calyptra and filament apex shape, presence of granules, and presence of unique traits (e.g., nodules). For each of the selected strains, 80 measurements of cell dimensions and 10 measurements of calyptra/filament apex were taken. Morphological analysis was described in depth in Skoupý et al. (2024). The morphology of the four herbarium specimens was studied separately under a Zeiss Axio Microscope (Zeiss, Germany) equipped with a MRc digital camera and 1000x magnification. For the four herbarium specimens, 200 measurements of cell length and cell width were taken in total (~50 measurements per sample), depending on the state of the preserved filaments.

### Analysis of anatoxin production in selected strains

Initial in silico scans were used to detect genes coding anatoxin production using the antiSMASH pipeline 6.0 (Blin et al., 2021) in all 201 genomes. If detected, liquid chromatography mass spectrometry/mass spectrometry (LC–MS/MS) analysis of toxins extracted from dried biomass was performed. Toxins were extracted from biomass by adding 200 μL of 0.1% acetic acid, followed by two cycles of freezing and thawing, followed by ultrasonication for 10 min (Fastner et al., 2023). The analysis was performed on an Agilent 2900 HPLC system (Agilent Technologies, Waldbronn, Germany) coupled to an API mass spectrometer 5500 QTrap (AB Sciex, Framingham, Massachusetts, United States) equipped with a turbo‐ion spray. Identification and quantification of anatoxins (anatoxin‐a, dihydro anatoxin‐a, homonantoxin‐a) were described in detail in Fastner et al. (2023).

### Taxonomy and nomenclature

We combined the monophyletic species concept sensu Johansen and Casamatta (2005), biological species concept sensu Mayr 1942 (modified by Dvořák et al., 2023 and Stanojković et al., 2024), and universal probabilistic concept of evolutionary lineages (UPCEL; Kollár et al., 2022) to erect new species. We followed the taxonomic workflow proposed in Dvořák et al. (2025). Geological data were obtained from the online database OneGeology (https://portal.onegeology.org) and soil information was obtained from the FAO‐UNESCO database (https://www.fao.org/soils‐portal/data‐hub/soil‐maps‐and‐databases) online database. The species descriptions conform to the rules of the International Code of Nomenclature for Algae, Fungi, and Plants (Turland et al., 2025). Names for new taxa follow Stearn (1983). Herbarium designations follow Thiers (2025).

## RESULTS

### Formal descriptions and taxonomic amendments

#### *Microcoleus vaginatus* (Vaucher) Gomont, 1892, (Figure 1a–d)

**FIGURE 1:**
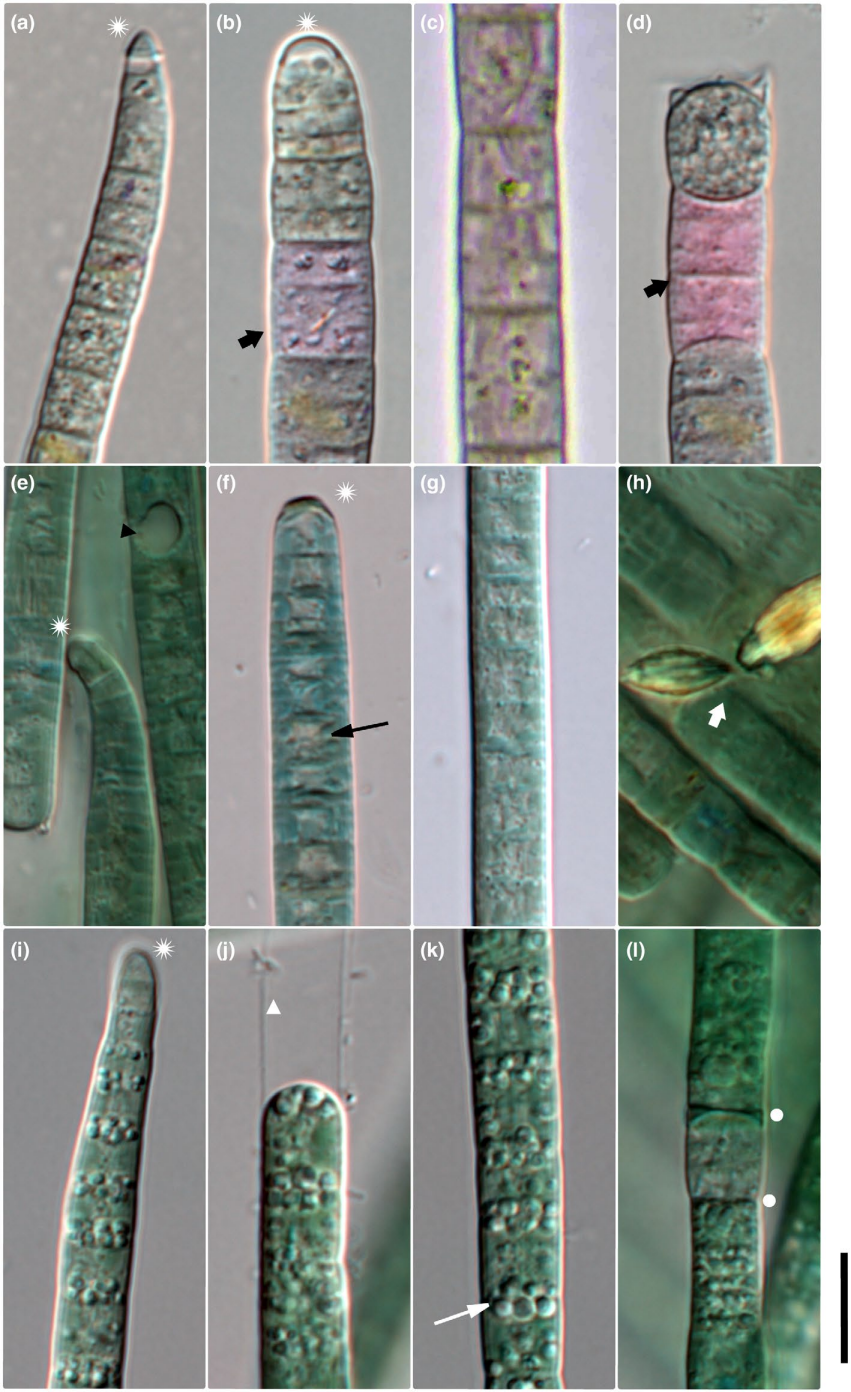
Microphotographs of *M. vaginatus* (a–d), *M. attenuatus* (e–h), and *M. pseudofavosus* (i–l). Scale = 10 μm, asterisk = calyptrate apical cell, circle = necridic cell, thick white arrow = extracellular crystals, thick black arrow = purple cells, thin white arrow = granules, and thin black arrow = cell content concentrated toward cell walls.

**FIGURE 2:**
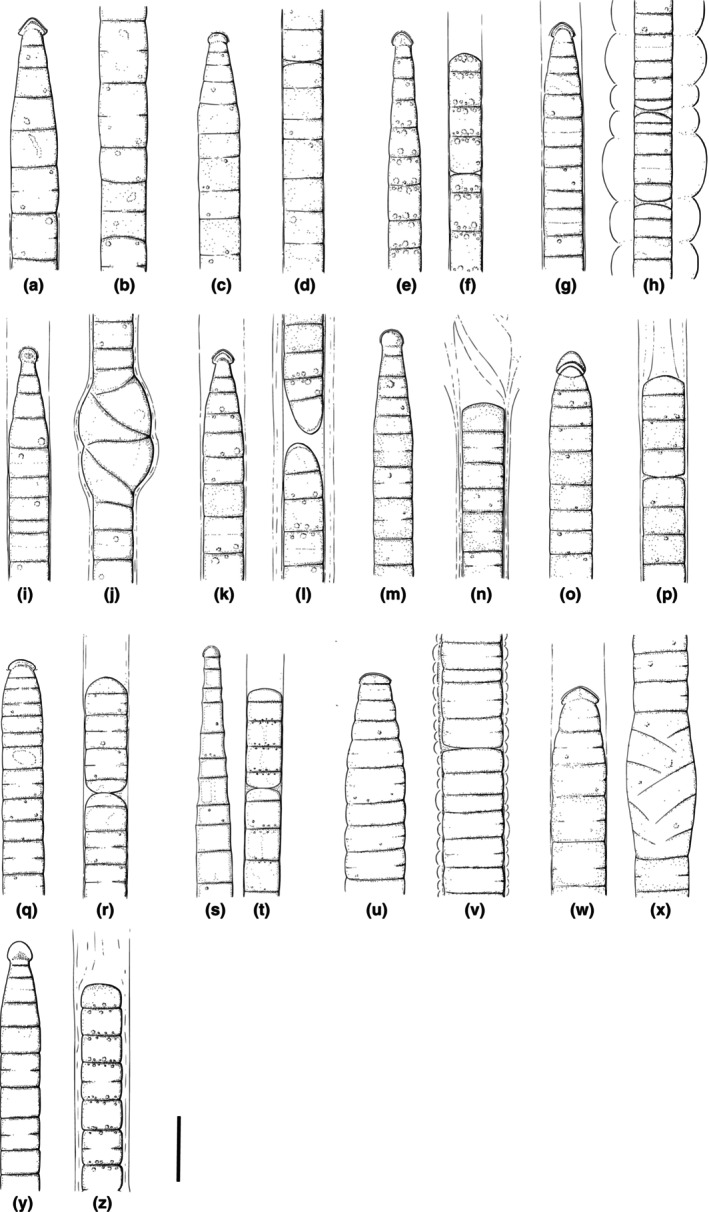
Lineworks of *M. vaginatus* (a, b), *M. attenuatus* (c, d), *M. pseudofavosus* (e, f), *M. ladakhensis* (g, h), *M. drouetii* (i, j), *M. harperi* (k, l), *M. toxifilus* (m, n), *M. pellucidapex* (o, p), *M. malodorus* (q, r), *M. tentaculus* (s, t), *M. atroviridis* (u, v), *M. crotalus* (w, x), and *M. occultus* (y, z). Scale = 10 μm.

***Amended description***: Filaments dark green or purple in color (Figure 1b,d). Sheaths mostly thin and indistinct or absent, with a single filament per sheath. Filaments often exceeding sheaths. Cells 3.5–9.55 μm wide (mean 6.96 μm) and 3.13–10.56 μm long (mean 6.27 μm), often isodiametric or longer than they are wide (Figures 1c and 2b). Trichomes cylindrical, gradually attenuating toward calyptrate apical cell (Figures 1a and 2a). Filament apex pointiness 0.11–0.99 (mean 0.37). Cell content with conspicuous small, often dense granules. Granules often concentrated toward distinct cross walls, with slight constrictions, and *Oscillatoria*‐like cell division (meristematic regions of consecutive cell divisions). Reproduction by necridic cells and subsequent breaking of the filament into hormogonia.

***Type locality*:** France

***Lectotype*:** BM_001213989, specimen from France, collected 1822 by Dezmazières, Pl. Crypt. France, ed. 1, no. 55. Designated in Drouet (1968, p. 228); ***Habitat*:** Damp soil, “En automne et au printemps, sur la terre nue de nos jardins, et sur les pots humides des plantes que nous y cultivons.” (“In autumn and spring, on the bare earth of our gardens and on the damp pots of the plants we grow there.”). From original herbarium collection by Dezmazières, Pl. Crypt. France.

***Epitype here designated in support of the above lectotype*:** OL_53201, a dried herbarium specimen of the reference strain C2_C6, collected 2019, Edge of an agricultural field, Czech Republic, GPS: 49.647133 17.368682

***Reference strain (ex‐epitype)*:** *Microcoleus vaginatus* C2_C6, deposited in the CCALA culture collection (Třeboň, Czech Republic). *GenBank Accession Numbers*: 16S rRNA (MW742832), ITS (MW754832) genome assembly (JAUBTP000000000)

***Comment*:** *Microcoleus vaginatus* formed a distinct monophyletic branch as a sister clade to *M. asticus* (Churro et al., 2020). *Microcoleus vaginatus* sensu stricto differed from *M. asticus* by the cell shape and dimensions as well as by the distinct dark green to purple coloration. *Microcoleus vaginatus* could be differentiated from *M. asticus* based on average nucleotide identity (ANI) value (92.2%) and distinct apomorphy in the form of cell shape and coloration,à and by phylogenomic analysis. *Microcoleus vaginatus* could be differentiated from other species by ANI value (Table 1, highest with *M. ladakhensis* at 92.12%), phylogeny, and morphology.

#### *Microcoleus attenuatus* (Fritsch), Strunecký, Komárek et Johansen (Figure 1e–h)

***Amended description*:** Filaments green to dark green, with mostly indistinct, sometimes distinct sheaths. Cells 3.95–8.64 μm wide (mean 6.3 μm) and 1.58–5.24 μm long (mean 3.03 μm). Trichomes cylindrical, attenuating gradually toward round calyptra (Figures 1e and 2c). Filament apex pointiness 0.08–0.74 (mean 0.30). Granules small, often concentrated at cross walls, cell content sometimes concentrated toward cell periphery (Figure 1f). Cross walls mostly distinct, sometimes indistinct, with slight constrictions. Reproduction by necridic cells and subsequent breaking of the filament into hormogonia (Figure 2d). Extracellular crystals sometimes present (Figure 1h).

***Type locality***: South Victoria Land, Antarctica

***Lectotype*:** F.E. Fritsch, no. 9 (British Museum), designated in Drouet (1968, p. 240, as *Lyngbya attenuata* Fritsch 1912). Collected December 1902. Melt water in freshwater pond among the lakes which border the shore of the Western Mainland; ***Habitat*:** Melt water in freshwater pond among the lakes that border the shore of the Western Mainland

***Epitype here designated supporting the above lectotype*:** OL_53202, a dried herbarium specimen of the reference strain A2_D2, collected 2019, growing among mosses on rocky coastal slopes of western Antarctica, GPS: −67.13342, −67.49444

***Reference strain (ex‐epitype)*:** *Microcoleus attenuatus* A2_D2, deposited in the CCALA culture collection (Třeboň, Czech Republic). *GenBank Accession Numbers*: 16S rRNA (MW742781), ITS (MW754772) genome assembly (JAUBVV000000000)

***Comment*:** The available morphological descriptions of *Microcoleus attenuatus* (Strunecký et al., 2013), formerly *Phormidium attenuatum* (Fritsch) Anagnostidis et Komárek (1988) fit the morphology of cultivated strains from the M11 clade, as did the general ecology, with captured occurrences of *M. attenuatus* from coastal Antarctica and Canadian Arctic (Strunecký et al., 2013). Furthermore, in the 16S rRNA gene phylogenetic tree (Figure S1), strains SVS1, KI‐19, KG29, and L12, denoted in Strunecký et al. (2013) as *M. attenuatus*, clustered with strains from clade M11. *Microcoleus attenuatus* formed a distinct and well‐supported monophyletic clade in the *Microcoleus* species continuum. *Microcoleus attenuatus* was morphologically very similar to *M. drouetii*, as the difference in cell dimensions was statistically insignificant (Skoupý et al., 2024); *M. attenuatus*, however, differed in the shape of the filament apex and thickness of sheaths, as *M. attenuatus* was not observed to have thick or layered sheaths or more than one filament per sheath when grown in culture. *Microcoleus attenuatus* can be safely recognized by ANI value (Table 1, highest with *M. crotalus* at 89.63%), genomic divergence, and phylogenomic analysis.

#### *Microcoleus pseudofavosus* sp. nov. Skoupý, Stanojković et Dvořák (Figure 1i–l)

***Description*:** Filaments blue green to gray green. Thin, distinct, colorless sheaths (Figure 1j); rarely do two filaments share a common sheath. Cells 4.07–6.43 μm wide (mean 5.01 μm) and 2.08–7.88 μm long (mean 4.14 μm), at times almost isodiametric. Trichomes are cylindrical, gradually attenuating toward calyptrate apical cells (Figure 1i; Figure 2e). Filament apex pointiness 0.04–0.59 (mean 0.22). Granules small, often concentrated at cross walls; larger granules sometimes present in cytoplasm (Figure 1k). Cross walls mostly distinct, with mild constrictions. Reproduction by necridic cells (Figures 1l and 2f) and subsequent breaking of the filament into hormogonia.

***Type locality*:** Slopes of coastal highlands, Western Svalbard (Norway), GPS: 78.536198, 15.929578

***Holotype*:** OL_53203, a dried specimen of the reference strain SVA1_A4, isolated from a well‐developed soil crust, collected 2019; **Habitat:** soil crust, slope deposits, Carboniferous‐permian marble bedrock, exposed Dryas tundra

***Reference strain (ex‐holotype)***: *Microcoleus pseudofavosus* SVA1_A4, deposited in the CCALA culture collection (Třeboň, Czech Republic). *GenBank Accession Numbers*: 16S rRNA (MW743034), ITS (MW755028) genome assembly (JAUBPE000000000)

***Comment*:** *Microcoleus pseudofavosus* formed a distinct, monophyletic and well‐supported clade among the *Microcoleus* species continuum. This species fit the morphological and ecological characteristics of the unrevised *M. favosus* as reported in Komárek and Anagnostidis (2005). Gomont used *Oscillatoria favosa* as the source name for *Phormidium favosum*, but the description of that species included 14 other taxa from diverse habitats all over Europe as well as in the United States, Guyana, and Australia. *Oscil. favosa* was collected from a hot spring in Belgium, but all other habitats listed were from various substrates in streams and waterfalls. Drouet (1968, p. 229) designated a lectotype for *O. favosa* based on Bory's material in PC collection, and so *P. favosum* and later *Microcoleus favosum* were lectotypified by Drouet's designation. Consequently, it seems unlikely that the clade we obtained contains populations that would be the same lineage as the hot spring taxon described by Bory, despite the fact that many GenBank accessions are attributed to *M. favosus* (Strunecký et al., 2013). We thus chose to describe this set of populations as *M. pseudofavosus*. This species could be differentiated from other species in the *Microcoleus* species continuum by the narrower filaments (mean 5.01 μm) with cells often isodiametric to even longer than they are wide (2.08–7.88 μm, mean 4.14 μm). Strains in this clade had distinct attenuation of the filament apex, different from either clade of our dataset. *Microcoleus pseudofavosus* was reliably recognized using the morphological characteristics, as well as by ANI value (Table 1, highest with *M. vaginatus* at 91.44%), genomic divergence, and phylogenomic analysis.

***Etymology*:** The species epithet was derived from *pseudo—*not genuine*—*and *favosus*—in reference to the unrevised *Microcoleus favosus*.

#### *Microcoleus ladakhensis* sp. nov. Skoupý, Stanojković et Dvořák (Figure 3a–d)

**FIGURE 3:**
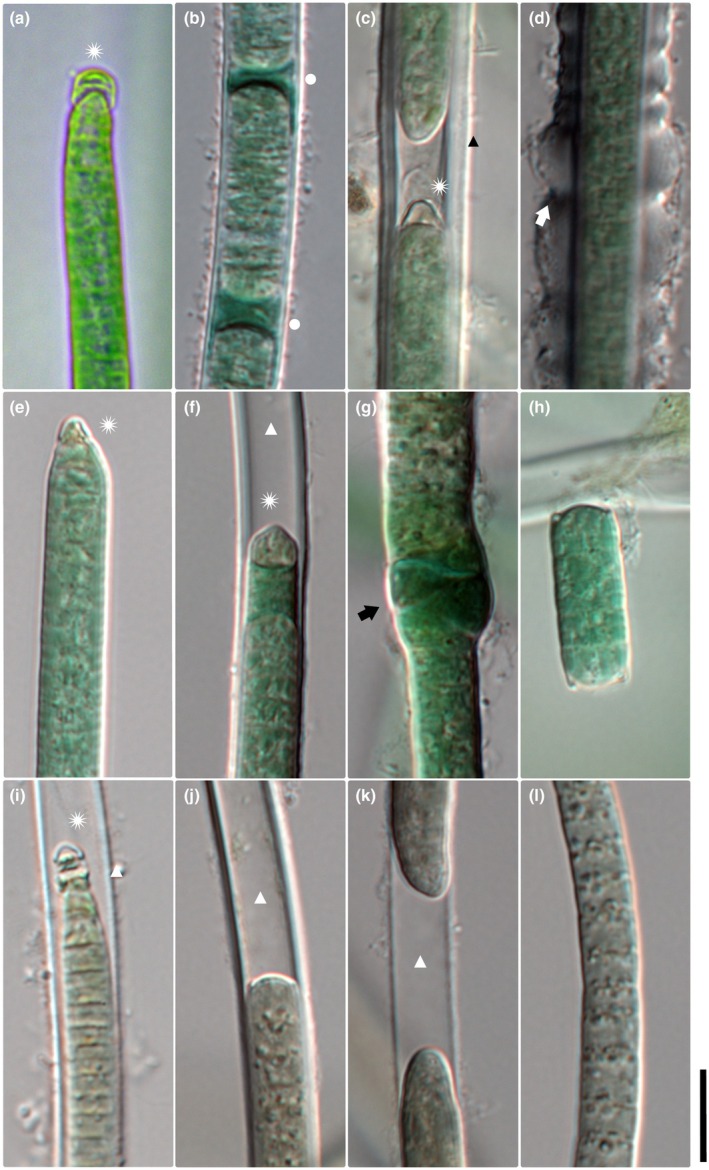
Microphotographs of *M. ladakhensis* (a–d), *M. drouetii* (e–h) and *M. harperi* (3i–l). Filament apices with calyptrate cell are marked by an asterisk. Scale = 10 μm, asterisk = calyptrate apical cell, triangle = empty sheath, black triangle = layered sheath, circle = necridic cell, white arrow = thick sheath with ring‐like constrictions, and black arrow = node.

***Description*:** Filaments light green to blue green or gray green in color. Distinct, often thin (sometimes thick) sheaths, which are occasionally layered (Figure 3c) and may exceed the filament (Figure 3c), sometimes with ring‐like constrictions (Figures 2h and 3d). Two or more filaments may share a common sheath (Figure 3c), nodules, or knots sometimes present. Cells 3.8–7.75 μm wide (mean 5.7 μm) and 1.05–5.19 μm long (mean 2.54 μm). Trichomes cylindrical, attenuating abruptly toward wide calyptra (Figures 3a and 2g). Filament apex pointiness 0.11–0.77 (mean 0.33). Cell directly under the calyptra rarely appears empty. Granules small and mostly sparse, often concentrated at cross walls. Cross walls mostly distinct (sometimes indistinct), with slight constrictions. Reproduction by necridic cells (Figure 3b; Figure 2h) and subsequent breaking of the filament into hormogonia.

***Type locality*:** Mountain slopes above Pare Chhu river, Ladakh (India), GPS: 32.59576, 78.26165

***Holotype*:** OL53204, a dried herbarium specimen of the reference strain Lad1_D1, isolated from a soil surface sample, collected 2019; **Habitat:** Soil surface, Eutric cambisol soil, Tanglangla geological formation, cold desert

***Reference strain (ex‐holotype)*:** *Microcoleus ladakhensis* Lad1_D1 deposited in the CCALA culture collection (Třeboň, Czech Republic). *GenBank Accession Numbers*: 16S rRNA (MW743191), ITS (MW755182) genome assembly (JAUBSC000000000).

***Comment*:** *Microcoleus ladakhensis* (M7) formed a distinct monophyletic clade among the *Microcoleus* species continuum, as a sister clade to *M. drouetii* and *M. harperi*. This species fit the general morphological characteristics of the unrevised species *Phormidium amoenum* as reported in Komárek and Anagnostidis (2005). However, *M. amoenus* (Kützing ex Gomont) Strunecký, Komárek et Johansen was reported from freshwater, muddy water, moist soils, water plants, flowing hot springs, and travertine, and originally described from a caldera in Lutetia (Paris) by Kützing. As the M7 clade is a soil clade, we considered use of *M. amoenus* as not a good fit for our taxon; *M. ladakhensis* was morphologically nearly cryptic to the M5 and M6 species, with the exception of cell dimensions. *Microcoleus ladakhensis* could be safely recognized using phylogenomic analysis, genome‐divergence, and ANI value (Table 1, highest with *M. harperi* at 93.49%).

***Etymology*:** *Microcoleus ladakhensis* was named after the geographic place of origin of the environmental sample, in Ladakh, India.

#### *Microcoleus drouetii* sp. nov. Skoupý, Stanojković et Dvořák (Figure 3e–h)

***Description*:** Filaments light green to blue green or gray green, rarely brown green in color, with distinct and sometimes thick sheaths (Figure 3f). Sheaths are occasionally layered and exceed the filament, sometimes spirally splitting. Two or more filaments may share a common sheath, with nodules or knots sometimes present (Figure 3g; Figure 2j). Cells 2.9–8.8 μm wide (mean 6.16 μm) and 1.07–6.9 μm long (mean 3.07 μm). Trichomes cylindrical, attenuating abruptly toward narrow calyptra (Figure 3e,f; Figure 2i). Filament apex pointiness 0.07–0.93 (mean 0.38). Granules small and mostly sparse, often concentrated at cross walls, cell content sometimes concentrated toward cell periphery. Cross walls mostly distinct, with slight constrictions. Reproduction by necridic cells and subsequent breaking of the filament into hormogonia (Figure 3h).

***Type locality*:** City of Columbia, Missouri, USA. GPS: 38.946382, −92.3143833 (approximate)

***Holotype*:** BM_001213995, a dried herbarium specimen deposited by Francis Drouet as *Microcoleus vaginatus*, collected October 11, 1938; **Habitat:** Surface of soil in a stone quarry, limestone bedrock

***Reference strain*:** *Microcoleus drouetii* AT8_B4 deposited in the CCALA culture collection (Třeboň, Czech Republic). *GenBank Accession Numbers*: 16S rRNA (MW742978), ITS (MW754985) genome assembly (JAUBUZ000000000)

***Comment***: *Microcoleus drouetii* (M5) formed a distinct monophyletic clade as a sister clade to *M. ladakhensis* and *M. harperi*. *Microcoleus drouetii* was morphologically cryptic to *M. persimillus* and nearly cryptic to *M. ladakhensis* with the exception of cell dimensions. *Microcoleus drouetii*, however, formed a distinct branch in the phylogenomic analysis as a sister clade to *M. harperi* and could be differentiated from said species by genomic divergence and ANI value (Table 1, highest with *M. harperi* at 93.39%).

***Etymology*:** *Microcoleus drouetii* was named in honor of a well‐known American phycologist, Francis Elliot Drouet (†1982), who contributed significantly to the understanding of filamentous cyanobacteria and was the author of many revisions and herbarium collections.

#### *Microcoleus harperi* sp. nov. Skoupý, Stanojković et Dvořák (Figure 3i–l)

***Description*:** Filaments light green to blue green or gray green (rarely brown green). Sheaths distinct, usually thin (sometimes thick), occasionally layered and exceeding filament (Figure 3i–k), sometimes spirally splitting. Two or more filaments may share a common sheath (Figures 3k and 2l). Nodules or knots occasionally present. Cells 3.0–8.5 μm wide (mean 6.0 μm) and 1.2–6.74 μm long (mean 3.15 μm). Trichomes cylindrical, abruptly attenuating toward narrow calyptra (Figures 3i and 2k). Filament apex pointiness 0.08–0.69 (mean 0.31). Granules small and mostly sparse, often concentrated at cross walls (Figure 3l). Cell content sometimes concentrated toward cell periphery. Cross walls mostly distinct, with slight constrictions. Reproduction by necridic cells and subsequent breaking of the filament into hormogonia.

***Type locality*:** Łódź Voivodeship, near the city of Piotrków Trybunalski, Poland. GPS: 51.307307, 19.714972

***Holotype*:** OL_53205, a dried herbarium specimen of the reference strain Pol14_C6, isolated from a soil sample, collected 2019; **Habitat:** Edge of a forest adjacent to an agricultural field, soil surface, gravel clays, deciduous forest

***Reference strain (ex‐holotype)*:** *Microcoleus harperi* Pol14_C6 deposited in the CCALA culture collection (Třeboň, Czech Republic). *GenBank Accession Numbers*: 16S rRNA (MW743186), ITS (MW755177) genome assembly (JAUBQN000000000)

***Comment*:** *Microcoleus harperi* was morphologically cryptic and virtually indistinguishable from *M. drouetii* and could be differentiated from *M. ladakhensis* solely by cell dimensions (Skoupý et al., 2024). *Microcoleus harperi*, however, formed a distinct branch in the phylogenomic analysis as a sister clade to *M. drouetii* and could be differentiated from said species by genomic divergence. *Microcoleus harperi* could be distinguished from *M. drouetii* using the genome‐divergence and ANI value (Table 1, highest with *M. ladakhensis* at 93.49%).

***Etymology*:** *Microcoleus harperi* was named in honor of Kimball Taylor Harper (†2011), an American researcher who greatly contributed to our understanding of the complex communities that are soil crusts. He coined the term cryptogamic crust and established the Brigham Young University school of biological soil crust biologists, including Samuel Rushforth, Jeffrey Johansen, Jayne Belnap, David Anderson, and Larry St. Clair.

#### *Microcoleus toxifilus* sp. nov. Skoupý, Stanojković et Dvořák (Figure 4a–c)

**FIGURE 4:**
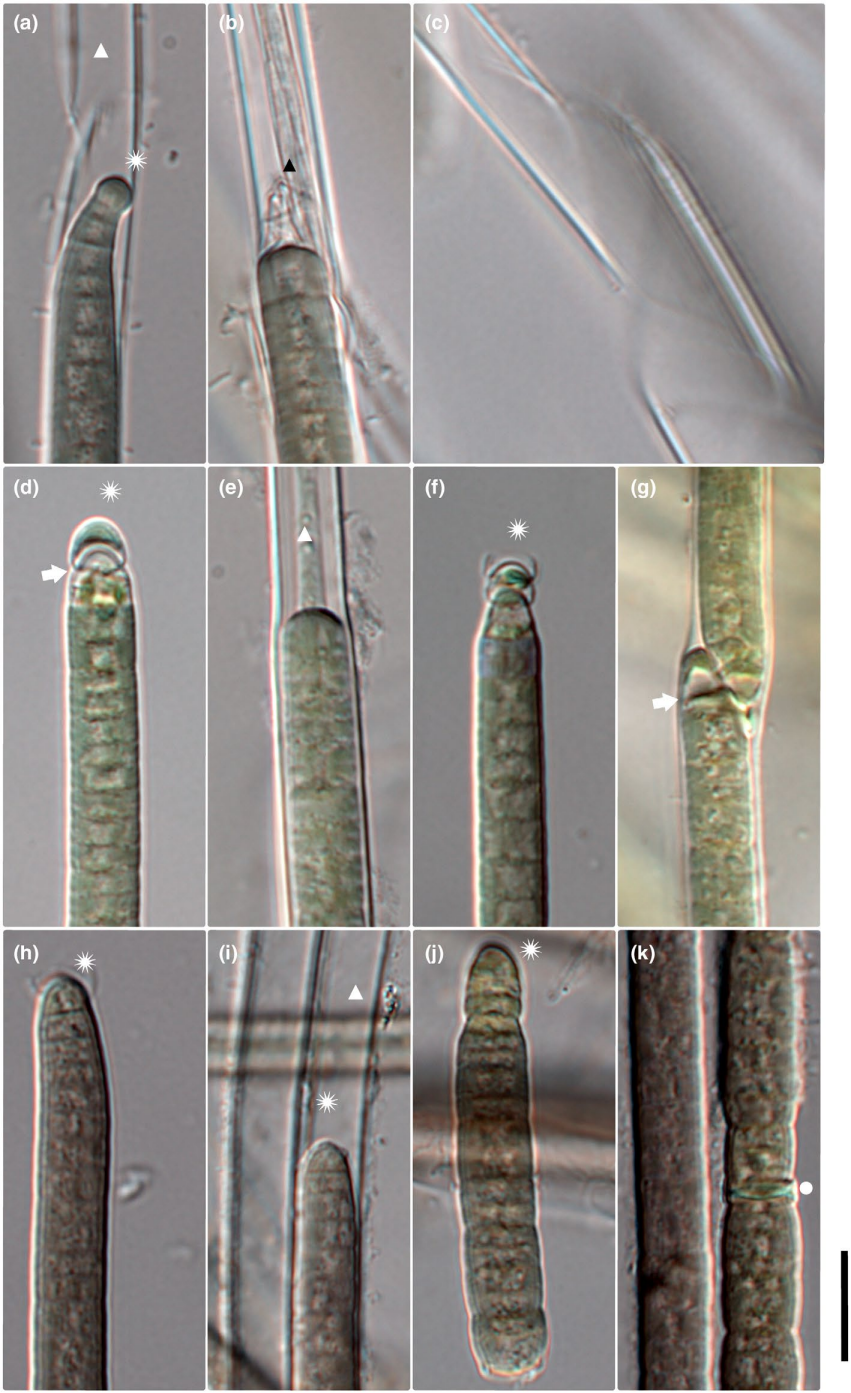
Microphotographs of *M. toxifilus* (a–c) *M. pellucidapex* (d–g), and *M. malodorus* (h–k). Scale = 10 μm, asterisk = calyptrate apical cell, triangle = empty sheath, black triangle = layered sheath, circle = necridic cell, and white arrow = empty cell bellow calyptra.

***Description*:** Filaments gray green to brown green (rarely dark green) in color. Distinct, very thick sheaths, often layered and exceeding filament, sometimes spirally splitting or with ring‐like constrictions (Figures 4b,c and 2n). Two or more filaments may share a common shealth; nodules, or knots occasionally present (similar to *Microcoleus drouetii*). Cells 2.6–8.37 μm wide (mean 6.28 μm) and 1.69–6.68 μm long (mean 3.18 μm). Trichomes cylindrical, attenuating abruptly toward wide, rounded, rarely layered, calyptra (Figures 4a and 2m). Filament apex pointiness 0.10–0.77 (mean 0.38). Cell content sometimes concentrated toward cell periphery, granules small and generally dense, often concentrated at cross walls. Cross walls distinct, with *Oscillatoria*‐like cell division (meristematic regions of consecutive cell divisions, Figure 2n) and slight constrictions. Reproduction by necridic cells and subsequent breaking of the filament into hormogonia. Genes for toxin production have been identified in this taxon.

***Type locality***: West Palm Beach, Florida, South‐Eastern USA. GPS: 26.690126, −80.060066

***Holotype*:** OL_53206, a dried herbarium specimen of the reference strain F8_C4, isolated from a soil sample, collected 2019. **Habitat:** moist soil surface, Pleistocene limestone bedrock, urban greenery.

***Reference strain (ex‐holotype)*:** *Microcoleus toxifilus* F8_C4; deposited in the CCALA culture collection (Třeboň, Czech Republic). *GenBank Accession Numbers*: 16S rRNA (MW743046), ITS (MW755040) genome assembly (JAUBSM000000000)

***Comment*:** *Microcoleus toxifilus* formed a stable and well‐supported branch in the phylogenomic tree and strains in this species possessed a unique apomorphy in the form of distinct, often thick, and even layered sheaths with ring‐like constrictions that often exceeded the filament itself and were, at times, spirally splitting and peeling away from the filament. This trait appeared to be unique, as we could not find any corresponding representation of *Microcoleus* (or *Phormidium*) with a spirally detaching sheath in available databases or literature. We also observed genes for anatoxin production in the genome of strains from this species and directly confirmed anatoxin‐a production in some of the strains (namely strains F8_D3, F8_D1, F8_C3, and F8_C1). *Microcoleus toxifilus* could be safely recognized by the distinct apomorphy, as well as by ANI value (Table 1, highest with *M. malodorus* at 90.03%), genomic divergence, phylogenomic analysis, and the production of anatoxin.

***Etymology*:** Species epithet *Microcoleus toxifilus* was derived from Latin *toxicus—*toxic—and *filum*—filament.

#### *Microcoleus pellucidapex* sp. nov. Skoupý, Stanojković et Dvořák (Figure 4d–g)

***Description*:** Filaments gray green to dark green in color. Sheaths mostly distinct, sometimes thick and layered (Figure 4e), may exceed filaments, and sometimes spirally splitting. Two or more filaments may share a common sheath (Figure 4g), with nodules or knots occasionally present. Cells 5.2–8.8 μm wide (mean 6.68 μm) and 1.8–4.52 μm long (mean 2.94 μm). Trichomes cylindrical, abruptly attenuating toward a calyptra (Figure 4d,f). Filament apex pointiness 0.10–0.88 (mean 0.46). The cell directly under the calyptra often appears empty (Figures 2o and 4d,f,g). Cell content sometimes concentrated toward cell periphery, granules small and generally dense, often concentrated at cross walls, which are distinct, with slight constrictions. Reproduction by necridic cells and subsequent breaking of the filament into hormogonia (Figure 2p).

***Type locality*:** City of Tønsberg, Southern Norway, GPS: 59.269359, 10.401542

***Holotype*:** OL_53207, a dried herbarium specimen of the reference strain N9_A3, isolated from a sample collected 2019. **Habitat:** Freshwater puddle, urban green space—dock on the fjord, Permian igneous bedrock, podsol soil

***Reference strain (ex‐holotype)***: *Microcoleus pellucidapex* N9_A3; deposited in the CCALA culture collection (Třeboň, Czech Republic). *GenBank Accession Numbers*: 16S rRNA gene (MW742940), ITS (MW754936) genome assembly (JAUBRF000000000)

***Comment*:** *Microcoleus pellucidapex* formed a distinct, monophyletic, and well‐supported clade in the *Microcoleus* species continuum. Strains of *M. pellucidapex* possessed an apomorphy in the form of seemingly empty cell directly under calyptra, which did not contain chromoplasm and thus appeared empty when viewed under a light microscope. *Microceleus pellucidapex* also differed distinctly in the shape of filament apex from other studied species of *Microcoleus* (Skoupý et al., 2024). *Microcoleus pellucidapex* can be safely recognized by the distinct apomorphy, as well as by ANI value (Table 1, highest with *M. ladakhensis* at 92.09%), genomic divergence, and phylogenomic analysis.

***Etymology*:** Species epithet *Microcoleus pellucidapex* was derived from Latin *pellucidus*—clear—and *apex*—apex for the clear apical cell at the end of trichomes.

#### *Microcoleus malodorus* sp. nov. Skoupý, Stanojković et Dvořák (Figure 4h–k)

***Description*:** Filaments dark green to brown green in color. Distinct and at times very thick or layered sheaths, with single filament (Figure 4i). Filaments often exceeding sheaths. Cells 4.79–7.88 μm wide (mean 6.42 μm) and 1.55–5.49 μm long (mean 2.89 μm). Trichomes cylindrical, abruptly attenuating toward calyptrate apical cell (Figure 4h,j,k; Figure 2q). Filament apex pointiness 0.19–0.83 (mean 0.41). Cell content with conspicuous small, often dense granules. Granules often concentrated toward distinct cross walls, with *Oscillatoria*‐like cell division (meristematic regions of consecutive cell divisions) and slight constrictions (Figure 2q,r). Reproduction by necridic cells (Figure 4k) and subsequent breaking of the filament into hormogonia. Strains in culture produce a distinct, unpleasant smell, reminiscent of damp clay, and pond sediment.

***Type locality*:** West Palm Beach, Florida, South‐Eastern USA, GPS: 26.690126, −80.060066.

***Holotype*:** OL_53208, a dried herbarium specimen of the reference strain F10_B6, isolated from a soil sample, collected 2019. **Habitat:** Soil surface, Pleistocene limestone bedrock, urban greenery.

***Reference strain (ex‐holotype)*:** *Microcoleus malodorus* F10_B6, deposited in the CCALA culture collection (Třeboň, Czech Republic). *GenBank Accession Numbers*: 16S rRNA (MW743182), ITS (MW755173) genome assembly (JAUBSZ000000000)

***Comment*:** *Microcoleus malodorus* formed a clearly defined and stable phylogenomic cluster within the *Microcoleus* species continuum and although it was morphologically rather similar to *M. pellucidapex*, it differed distinctly in the shape of the filament apex. Strains in this clade also emitted a distinct, unpleasant smell reminiscent of damp clay and pond sediment when grown in culture. *Microcoleus malodorus* could be safely recognized by the shape of filament apex as well as by ANI value (Table 1, highest with *M. toxifilus* at 93.03%), genomic divergence, phylogenomic analysis, and the distinct smell.

**Etymology:** Species epithet *Microcoleus malodorus* was derived from Latin *malus*—bad—and *odor—*smell, due to the distinct unpleasant smell produced by strains in culture.

#### *Microcoleus tentaculus* sp. nov. Skoupý, Stanojković et Dvořák (Figure 5a–d)

**FIGURE 5:**
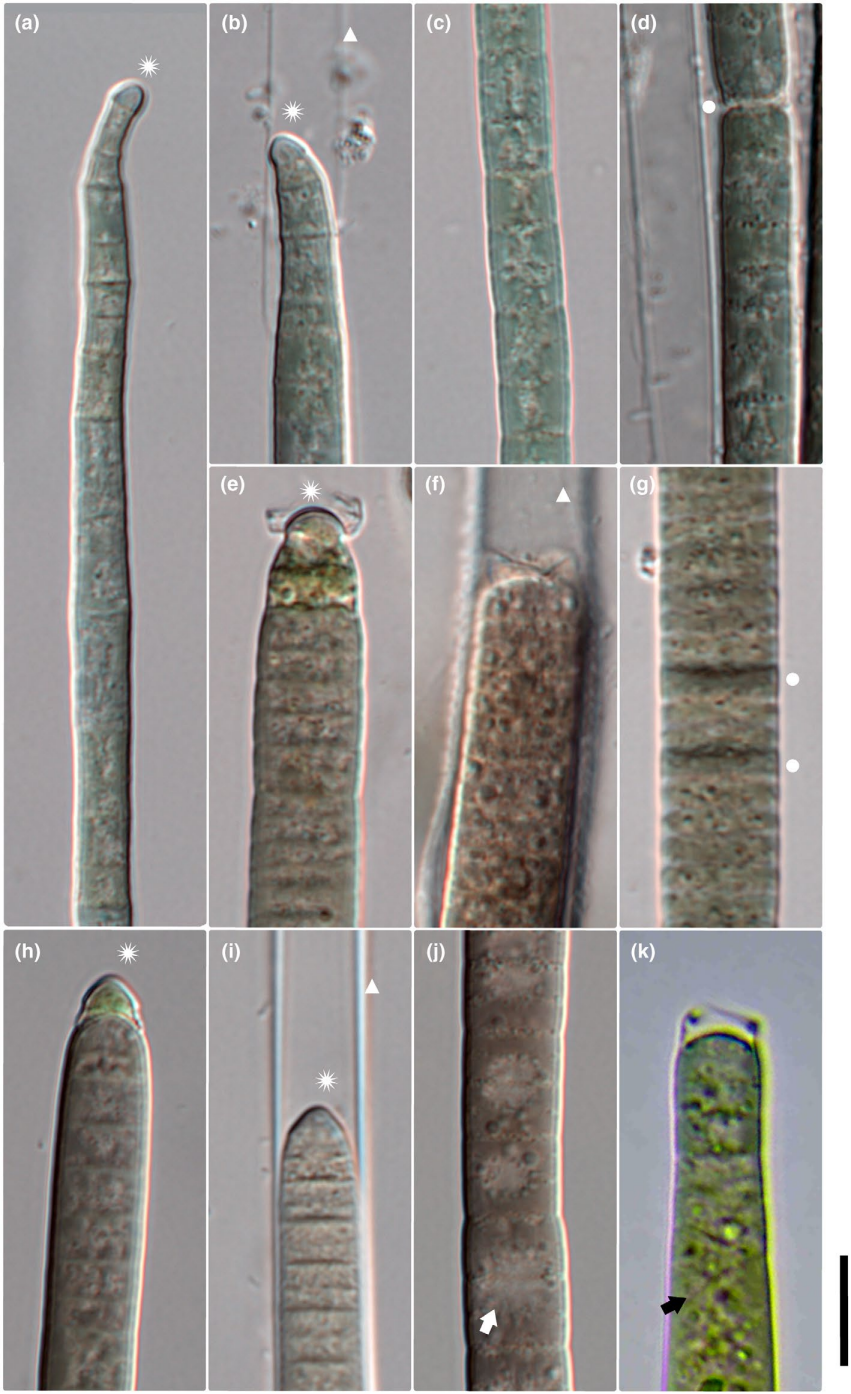
Microphotographs of *M. tentaculus* (a–d), *M. atroviridis* (e–g), and *M. crotalus* (h–k). Scale = 10 μm, asterisk = calyptrate apical cell, triangle = empty sheath, circle = necridic cell, white arrow = cell content concentrated toward cell walls, and black arrow = node of twisting cells.

***Description*:** Filaments light green to gray green. Sheaths thin, colorless, and indistinct, with a single trichome per sheath (Figure 5b,d). Cells 2.28–7.46 μm wide (mean 5.89 μm) and 1.64–7.91 μm long (mean 3.54 μm), at times almost isodiametric (Figure 5c). Trichomes cylindrical, very gradually attenuating toward calyptrate apical cell (Figures 2s and 5a,b). Filament apex pointiness 0.04–0.65 (mean 0.21). Cell content sometimes concentrated toward cell periphery, granules small and mostly sparse, often concentrated at cross walls (Figure 5d). Cross walls mostly distinct, without visible constrictions. Reproduction by necridic cells and subsequent breaking of the filament into hormogonia (Figure 2t).

***Type locality*:** City of Uppsala, South‐Eastern Sweden, GPS: 59.838631, 17.65673

***Holotype*:** OL_53209, a dried herbarium specimen of the reference strain S36b_C1, isolated from a soil crust sample, collected 2019. **Habitat:** Well‐developed soil crust, Precambrian gneissic bedrock, postglacial clays, urban greenery

***Reference strain (ex‐holotype)*:** *Microcoleus tentaculus* S36b_C1; deposited in the CCALA culture collection (Třeboň, Czech Republic). *GenBank Accession Numbers*: 16S rRNA (MW742740), ITS (MW754741) genome assembly (JAUBPM000000000)

***Comment*:** *Microcoleus tentaculus* formed a distinct and well‐supported branch in the *Microcoleus* species continuum. *Microcoleus tentaculus* also possessed a unique morphological apomorphy in the form of the long and gradual attenuation of the filament apex. This trait appeared to be quite unique, as we found no similar depiction of *Microcoleus* species in the available literature. Similar in morphology is *M. beggiatoiformis* (Strunecký et al., 2013) or the former *Phormidium beggiatoiforme* (Gomont) as depicted in Anagnostidis and Komárek (1988), which was described, however, as occupying a distinct ecological niche in the form of calcareous waters and travertine springs, dissimilar to any of the cultivated strains in our dataset. *Microcoleus tentaculus* could be safely recognized by the distinct apomorphy, as well as by ANI value (Table 1, highest with *M. attenuatus* at 89.4%), genomic divergence, and phylogenomic analysis.

***Etymology*:** Species epithet *Microcoleus tentaculus* was derived from Latin *tentaculum*—tentacle, for the distinct and gradual attenuation of the filament apices.

#### *Microcoleus atroviridis* sp. nov. Skoupý, Stanojković et Dvořák (Figure 5e–g)

***Description*:** Filaments dark green in color. Sheaths thin, colorless, and mostly indistinct (rarely distinct or thick and structured, Figure 5f; Figure 2v); single trichome per sheath. Cells 7.5–11.0 μm wide (mean 9.31 μm) and 1.8–9.83 μm long (mean 2.8 μm). Trichomes are cylindrical, abruptly narrowing toward apical cell with wide calyptra (Figures 2u and 5e). Filament apex pointiness 0.3–0.89 (mean 0.59). Granules small and mostly sparse (Figure 5f). Cross walls very distinct, with *Oscillatoria*‐like cell division. Slight constrictions at cross walls. Reproduction by necridic cells (Figure 5g) and subsequent breaking of the filament into hormogonia.

***Type locality*:** Forested area of the Lamington National Park, Eastern Australia, GPS: −28. 2230653, 153.1425189

***Holotype*:** OL_532010, a dried herbarium specimen of reference strain Aus8_D4, isolated from a sample collected 2019. **Habitat:** Surface of rock, tertiary mafic volcanic bedrock, coastal rainforest

***Reference strain*:** *Microcoleus atroviridis* Aus8_D4; deposited in the CCALA culture collection (Třeboň, Czech Republic). *GenBank Accession Numbers*: 16S rRNA (MW743019), ITS (MW754808) genome assembly (JAUBUL000000000)

***Comment*:** *Microcoleus atroviridis* formed a well‐defined, monophyletic branch within the *Microcoleus* species continuum. Strains from this species also possessed the widest trichomes in our dataset, at a mean width of 9.31 μm. This characteristic, together with the distinctive *Oscillatoria*‐like cell division and dark‐green color of filaments, distinguished this species from others in our dataset. *Microcoleus calidus* (Strunecký et al., 2013), formerly *Phormidium calidum* (Gomont, 1892), was morphologically relatively similar to *M. atroviridis*, but it differed significantly in ecology. A similar case is *M. rushforthii* (Casamatta et al., 2005), which had similar cell dimensions but differed in ecology. Furthermore, 16S rRNA phylogeny placed *M. rushforthii* neatly in the vicinity of *M. anatoxicus* (Conklin et al., 2020). *Microcoleus atroviridis* could be recognized by the distinct morphology, ANI value (Table 1, highest with *M. crotalus* at 90.79%), genomic divergence, and phylogenomic analysis.

***Etymology*:** Species epithet *Microcoleus atroviridis* was derived from Latin *atroviridis—dark* green, for the dark green color of filaments produced in culture.

#### *Microcoleus crotalus* sp. nov. Skoupý, Stanojković et Dvořák (Figure 5h–k)

***Description*:** Filaments gray green to brown green. Sheaths thin but distinct, with a single trichome per sheath. Sheaths often exceed filaments (Figure 5i). Cells 6.4–9.94 μm wide (mean 8.24 μm) and 1.64–11.3 μm long (mean 4.44 μm), at times isodiametric or longer than wide. Trichomes are cylindrical, abruptly narrowing toward the apical cell with a wide, sometimes layered, calyptra (Figure 2w). Filament apex pointiness 0.22–0.96 (mean 0.45). Segments of twisted cells of varying length are sometimes present (Figure 5k; Figure 2x). Cell content is sometimes concentrated toward the cell periphery (Figure 5j), granules small and mostly sparse, rarely concentrated at cross walls. Cross walls are distinct, with slight constrictions. Reproduction by necridic cells and subsequent breaking of the filament into hormogonia.

***Type locality*:** Southern Arizona, near Burro Canyon, Southern USA, GPS: 31.41316, −110.90755

***Holotype*:** OL_53211, a dried herbarium specimen of the reference strain ARI1_A2, isolated from a soil crust sample, collected 2019. **Habitat:** Roadside along a sparsely vegetated semi‐desert, well‐developed soil crust, Jurassic granitic bedrock, thermic semiarid soils

***Reference strain (ex‐holotype)*:** *Microcoleus crotalus* ARI1_A2; deposited in the CCALA culture collection (Třeboň, Czech Republic). *GenBank Accession Numbers*: 16S rRNA (MW742719), ITS (MW754729) genome assembly (JAUPTI000000000)

***Comment*:** *Microcoleus crotalus* formed a distinct, well‐supported monophyletic clade among the *Microcoleus* species continuum. *Microcoleus crotalus* also possessed a unique morphological trait in the form of nodes of spirally twisted cells. This trait appeared to be unique among the *M. vaginatus* group, as it was not observed in any other clade within our dataset. We were also unable to find any species matching the morphological characteristics of this putative species in available literature. *Microcoleus crotalus* could be safely recognized by the distinct apomorphy, as well as by ANI value (Table 1, highest with *M. atroviridis* at 90.79%), genomic divergence, and phylogenomic analysis.

***Etymology*:** The species name *Microcoleus crotalus* was derived from the Latin name for rattlesnakes—*Crotalus—*due to the similarity of the twisted nodes to the shape of a rattlesnake's tail.

#### *Microcoleus occultus* sp. nov. Skoupý, Stanojković et Dvořák (Figure 6a–d)

**FIGURE 6:**
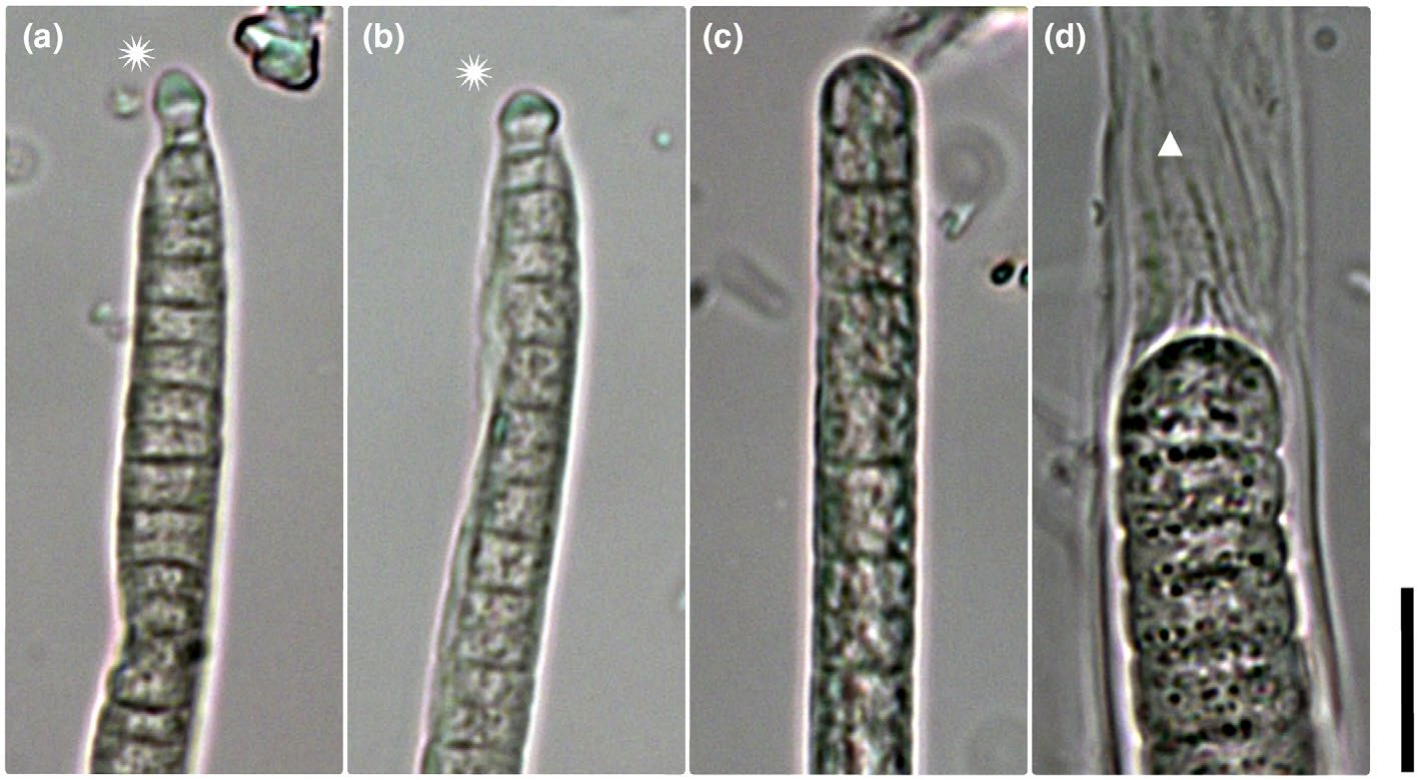
Microphotographs of *M. occultus*. Scale = 10 μm, asterisk = calyptrate apical cell, triangle = empty sheath.

***Description*:** Thallus very dark green, nearly black when dried. Filaments light green to gray green in color (in desiccated sample), straight or flexuous, with distinct, colorless sheaths. Cells 3.86–9.33 μm wide (mean 6.19) and 0.64–5.94 μm long (mean 3.09). Trichomes cylindrical, briefly attenuating toward apical cell with pronounced calyptra (Figures 2y and 6a,b). Cross walls distinct with mild constrictions (Figure 6c) and *Oscillatoria*‐like cell division (meristematic regions of consecutive cell divisions). Cells frequently granulated, with granules often concentrated at cross walls (Figures 2z and 6d).

***Type locality*:** City of Aberdeen, South Dakota, Northern USA, GPS: 45.4645, −98.4874 (approximate)

***Holotype*:** BM_001215583, a dried herbarium specimen, collected 1896. **Habitat:** In tank of artesian waters, heterogeneous sand and gravel of glaciofluvial origin

***Reference genome*:** M. occultus BM001215583, *GenBank* Accession Number: genome assembly (JAUBOG000000000)

***Comment*:** *Microcoleus occultus* formed a distinct, monophyletic, and well‐supported clade among the *Microcoleus* species continuum, comprised of four herbarium‐derived genomes from the herbarium collection of the Natural History Museum in London. The original herbarium samples were designated as *Phormidium uncinatum* (BM001215583), *P. subfuscum* (BM001215508 and BM001215366), and *M. vaginatus* (BM001213992).

**Etymology:** Species epithet *Microcoleus occultus* was derived from Latin *occultus—*hidden, since there are herbarium specimens of this species but no known living strain.

### Delineation of species within the *Microcoleus* speciation continuum

Bayesian hierarchical clustering revealed 21 distinct, well‐supported clades (M1 to M21). For delimitation of most species, the monophyletic species concept sensu Johansen and Casamatta was applicable, with the exception of for *Microcoleus ladakhensis*, *M. drouetii*, and *M. harperi*, which were morphologically cryptic and subjected to ongoing gene‐flow. The biological species concept was applicable to all, as the gene‐flow was higher within species than between species. The analysis of gene‐flow, genomic divergence, recombination, and pangenome was performed in Stanojković et al. (2024). The mean genome fraction subjected to gene flow between strains of the same species varied from 15% to 53.2% (within the lineages), whereas the genome fraction affected by gene flow from outside the species varied from 1.54% to 29.04% (Stanojković et al., 2024). The frequency of homologous recombination was higher within species than between them (Stanojković et al., 2024) and differed depending on the habitat preference of species. The pangenome contained 133,737 genes, with the core genome comprising only 0.54% of the pangenome (639 genes). The genomic divergence was estimated using overlapping 50‐kb sliding windows spanning the genome, and the mean absolute divergence at the interspecific level spanned 0.011–0.017. The mean fixation index (*F*_ST_) ranged from 0.20 to 0.93, which implies that lineage pairs represent distinct stages across the divergence continuum of *Microcoleus*, from early to late stages of speciation (Skoupý et al., 2024; Stanojković et al., 2024). The pairwise ANI showed that cultivated *Microcoleus* sp. strains shared 86.94%–99.9% sequence identity across their genomes, with ANI value between putative species being lower than the 95% identity threshold (Table 2) with most pairs having an ANI value below 90%, with the lowest shared identity being 87.12%, between species M1 and M4 (*M. atroviridis* and *M. toxifilus*, respectively).

**TABLE 2:** Mean ANI values (%) in species pairs.

	Mean ANI values											
1. *Microcoleus vaginatus*	**94.24**											
2. *M. attenuatus*	87.62	**97.48**										
3. *M. pseudofavosus*	91.45	88.26	**97.84**									
4. *M. ladakhensis*	92.12	87.74	91.26	**95.67**								
5. *M. drouetii*	91.87	87.52	91.01	93.07	**94.77**							
6. *M. harperi*	91.91	87.54	91.08	93.50	93.39	**94.68**						
7. *M. toxifilus*	91.39	87.42	87.84	92.46	92.80	92.69	**96.78**					
8. *M. pellucidapex*	91.01	89.17	90.53	90.97	91.94	92.06	91.28	**97.33**				
9. *M. malodorus*	91.82	87.62	91.43	92.10	92.06	92.27	93.03	90.97	**97.75**			
10. *M. tentaculus*	87.60	89.43	88.27	87.63	87.61	87.57	87.84	89.22	87.74	**98.15**		
11. *M. atroviridis*	87.30	89.51	87.90	87.34	87.17	87.16	87.12	87.80	87.39	87.59	**99.98**	
12. *M. crotalus*	88.99	89.64	89.41	88.90	88.74	88.82	88.70	88.93	88.98	88.40	90.80	**99.99**
	**1**	**2**	**3**	**4**	**5**	**6**	**7**	**8**	**9**	**10**	**11**	**12**

*Note*: Values lower than 90 are marked in red, intra‐species mean ANI values are marked in bold.

**FIGURE 7:**
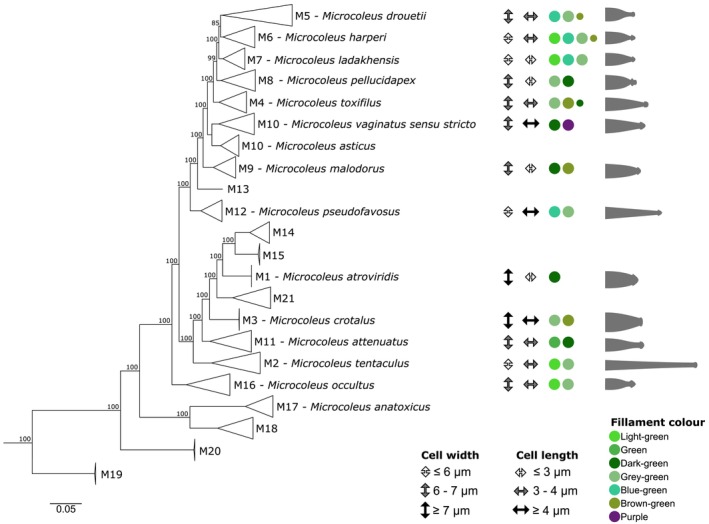
Whole‐genome phylogeny of *Microcoleus vaginatus* speciation continuum with morphology overview. Morphological values shown include cell dimensions, color of the filaments, and shape of the filament apices. The average cell size values are divided into three categories, indicated by double arrows. Colored circles indicate the color of filaments; smaller circles indicate lower frequency. The uncollapsed phylogenomic tree can be found in the Figure S2.

Of the 201 cultivated strains, 180 were morphologically analyzed in Skoupý et al. (2024). Although exhibiting *Microcoleus vaginatus*‐like morphology, distinct differences in cell dimensions, filament color, and the shape of the filament apex were seen among the studied clades, serving as potential apomorphies. We have provided here an overview of the morphological characteristics of morphologically studied putative species (Figure 7). The putative species M13, M14, M15, M17, M18, M19, M20, and M21 were either comprised of a single strain or comprised solely of metagenomes and were thus omitted from morphological analysis. The clade M16 consisted solely of a herbarium specimen, but we were able to obtain enough data for morphological analysis in the form of microphotographs of the herbarium specimen and historic observations and descriptions, and we elected to erect it as a novel species, *M. occultus*.

Furthermore, 16S rRNA gene phylogenetic analysis was performed (Figure S2). The 16S rRNA gene phylogeny proved to be insufficient for species articulation for *Microcoleus vaginatus* species continuum, as the 16S rRNA gene sequences did not cohere to the same topology as the whole‐genome phylogeny and were dispersed in the phylogenetic tree without any noticeable pattern past the major branch division. The 16S rRNA gene marker is thus not usable for taxonomic resolutions in the *Microcoleus* species continuum, as the diversity within this marker does not reflect the true diversity of the *M. vaginatus* group. Similarly, the use of ITS rRNA region secondary structures proved to be complicated in the case of the *Microcoleus* species continuum, as some species had distinct ITS rRNA region structures, whereas others had two or more types of ITS rRNA region structures (described in detail in Skoupý et al., 2024).

The in silico presence of anatoxin‐a genes was detected in nine of the cultivated strains of *Microcoleus* by antiSMASH analysis, four of which belonged to *M. drouetii* (M5), and five belonged to *M. toxifilus* (M4). Anatoxin‐a and its derivatives were directly detected in four strains, all of which belonged to *M. toxifilus* (M4). The table of anatoxin‐producing strains with detected concentrations can be found in Table S1.

## DISCUSSION

The taxonomy of Cyanobacteria is a dynamic and continually evolving field. However, due to high levels of cryptic diversity and historic reliance on morphology in delineation of new species, many species descriptions need revision in order to accurately reflect evolutionary relationships (Dvořák et al., 2023; Johansen et al., 2021; Komárek et al., 2020; Skoupý et al., 2024). Many species are considered taxonomically valid yet have not been recorded since their descriptions. Other species were amended by later authors, and those descriptions may be contradictory or incomplete (Kaštovský, 2024). Furthermore, the concept of what constitutes a Cyanobacterial species has evolved significantly over the past century, and taxonomists continue to debate the best approach for species delineation (Johansen & Casamatta, 2005). Many such species concepts exist, none of which are without caveats or limitations (Dvořák et al., 2023; Johansen & Casamatta, 2005; Kollár et al., 2022; Skoupý et al., 2024). Different groups of Cyanobacteria have differing rates of evolution and genetic coherence, and to bring Cyanobacterial taxonomy fully into the 21st century and make species delimitations as “close to natural” as possible, it may be necessary to break away from similarity‐based matrices and embrace population‐genomics‐based species data (Dvořák et al., 2023).

In our previous work (Dvořák et al., 2023), we developed and formulated a population‐genomics‐based approach of species descriptions, employing large‐scale population‐genomics datasets, combined with morphological data and herbarium‐derived data, to delineate Cyanobacterial species. This approach was subsequently employed on a dataset of 201 *Microcoleus* sp. genomes from cultivated strains, 100 metagenomes, and eight herbarium‐derived genomes (Skoupý et al., 2024). Combining phylogenomic, whole‐genome, and morphological analyses, we uncovered at least 21 putative species of *M. vaginatus‐*like taxa existing along a speciation continuum and exhibiting a continuum of morphological features. The 21 studied species also exhibited a range of biogeographic and ecological distributions, from species with limited geographic distribution to species with nearly cosmopolitan distributions (Skoupý et al., 2024; Stanojković et al., 2024).

In an effort to match our putative species to valid, existing ones, we searched available public sequence databases and previous revisions of the *Microcoleus*. We focused on the species in the *M. vaginatus* group (Komárek & Anagnostidis, 2005) and the *Phormidium* VII group (Komárek & Anagnostidis, 2005; Strunecký et al., 2013). These taxa are in the intermediate to thick group (3–10 μm wide filaments) and have calyptrate apical cells, considered an apomorphy for the *M. vaginatus* group. However, since these taxa have undergone many revisions, it was difficult to find concrete information on the morphology and ecology of these species.

The first issue that bears addressing is the phylogenetic position of the type species, *Microcoleus vaginatus*. The latest revision of *Microcoleus* proposed six distinct species based on 16S rRNA gene and ITS rRNA region phylogeny (Strunecký et al., 2013). Although Strunecký et al. (2013) proposed that *M. vaginatus* sensu stricto lay somewhere within one of these proposed clades (likely clade A), they did not set a new epitype or reference genome, which we sought to rectify here. Notably, strains from the clades A and B (Strunecký et al., 2013) clustered with strains from our dataset, specifically within the larger branch, from M12 (*M. pseudofavosus*) to M5 (*M. drouetii*). We note this implies that *M. vaginatus* lies somewhere within this branch based on a 16S rRNA gene phylogenetic tree (Figure S1).

Here, herbarium‐derived genomes provided us with an answer, as one of these historic genomes belonged to *Microcoleus terrestris* (BM001213989) collected by J.B. Desmaziéres in 1822. This herbarium sample was, perhaps, the oldest available herbarium specimen of *Microcoleus*. In the phylogenomic analysis, this strain clustered neatly into clade M10 with extant strains collected from Czech Republic and formed a sister clade to *M. asticus* (Churro et al., 2020). Notably, based on genomic divergence and ANI value alone, both clades (*M. asticus* and the clade containing *M. terrestris* BM001213989) would belong to the same species, but they could be safely distinguished in combination with phylogeny and distinct morphology (Skoupý et al., 2024). *Microcoleus* was originally erected by Desmaziéres with the type *M. terrestris*, later synonimized with *M. vaginatus* by Gomont (1892) who considered the original herbarium specimen by Desmaziéres as type material. Thus, we believe it is warranted to delineate this clade as *M. vaginatus* sensu stricto and set the original herbarium specimen collected by Desmaziéres as a type once more, returning *Microcoleus* to its roots.

*Microcoleus vaginatus* has long been considered a cosmopolitan and an ecologically significant species, making it the subject of a wide range of research, from ecology to biotechnology (Couradeau et al., 2019; Stanojković et al., 2022; Wang et al., 2025). With the retypification of this important species, however, we have caused a new issue—much of the earlier work likely did not examine *M. vaginatus* sensu stricto but, instead, one of several related or morphologically similar species within the *Microcoleus* speciation continuum, most likely *M. drouetii* or *M. harperi*, which appeared to have the broadest geographic distributions in our dataset. This somewhat challenges the view of *M. vaginatus* as a cosmopolitan, ecologically significant species. That being said, we believe that the retypification of *M. vaginatus* is supported by a robust dataset. Moreover, the characterization of the *Microcoleus* species continuum and the related species taxa that comprise it has the potential to refine our understanding of this ecologically significant group and its role in global ecosystems and to improve our understanding of this ecologically important group and the role that it plays in the world's ecosystems. Although *M. vaginatus* sensu stricto may not be as ubiquitous as previously believed, the broader *Microcoleus* species continuum remains a cosmopolitan and ecologically important assemblage that warrants continued study. Furthermore, although *M. vaginatus* sensu stricto itself might not be as ubiquitous as previously suspected, the *M. vaginatus* species continuum is still an important cosmopolitan Cyanobacterial group worth studying.

Notably, there are other unrevised species of *Microcoleus* in available literature with reported violet or purple colors similar to *M. vaginatus* sensu stricto, namely *M. setchelianus* (Strunecký et al., 2013), *M. walrothii* (Strunecký et al., 2013), and *M. violaceus* (Frémy 1930). However, no molecular data are available for these species. Although including these “purple” species in *M. vaginatus* sensu stricto on the basis of superficially similar morphology would help reduce the number of unrevised species, we believe this group deserves a deeper study, and for now, their taxonomic status remains unclear.

An interesting taxonomic issue was posed by the species M5 (*Microcoleus drouetii*), M6 (*M. harperi*), and M7 (*M. ladakhensis*). They appeared to be diverging species, with high levels of cryptic morphology and ongoing gene‐flow (Skoupý et al., 2024; Stanojković et al., 2024). Although morphologically very similar, they could be separated based on 16S rRNA gene phylogeny, genomic divergence, and ANI value. Furthermore, they could be separated via the biological species concept (adapted to prokaryotes) because the gene flow was more frequent within species than between species (Bobay, 2020; Dvořák et al., 2023; Stanojković et al., 2024). The three species could be thus delineated as novel species, morphologically cryptic and not yet fully diverged from each other. These species provided an opportunity to exercise defining cryptic species in the gray zone of ongoing speciation using the UPCEL, which allows conceptualization of the species boundaries in a probabilistic framework (see Kollár et al., 2022 for details). The three species had 73% of their genomes resistant to the gene flow (Stanojković et al., 2024), meaning a 73% probability of the species divergence. Thus, UPCEL helped us to recognize the phase of divergence among the cryptic species. It is likely that with time these three clades will fully diverge, possibly developing distinct apomorphies, but as of now, they can be delineated solely based on genome divergence and gene flow.

The M5 (*Microcoleus drouetii*) species was the most numerous clade in our dataset, being comprised of 81 strains with a wide geographic range. This species also contained the herbarium‐derived genome of *M. vaginatus* (BM001213995) collected by Drouet in 1938. We erected this putative species as a novel species, *M. drouetii*, with the herbarium specimen collected by Drouet (BM001213995) being set as type material. Similarly, the putative species M6, comprised of strains originating from Poland, Czech Republic, and Montana (United States), could be erected as the novel species *M. harperi*. The last of the trio of species, M7, comprised of strains from Ladakh (India) and Sweden, was erected as the novel species *M. ladakhensis*.

The M11 clade was a morphological and ecological fit for *Phormidium attenuatum* (sensu Anagnostidis & Komárek, 1988). Strunecký et al. (2013) revised *P. attenuatum* to *Microcoleus attenuatus* based on four cultivated strains from coastal Antarctica. The strains obtained by Strunecký et al. (2013) came from terrestrial habitats associated with high nitrogen and phosphorus enrichment (whale bones and bird rookeries), whereas our cultivated strains were associated with moss, suggesting a wider ecological range.

Species M12 matched the morphological description of *Microcoleus favosus* (as reported in Komárek & Anagnostidis, 2005) but was ecologically very different. *Microcoleus favosus* was mostly considered a freshwater species, isolated from cold flowing waters. However, *M. favosus* was a revision of *Oscillatoria favosa* (Bory, 1827), collected from a thermal spring in Belgium. This species was subsequently revisited by Gomont (1892b), who designated it as *Phormidium favosum* and included 14 other taxa. These taxa originated from varied habitats, climates, and geographic locations throughout the world, including Australia, Guayana, and the United States of America, introducing a rather large disparity in both ecology and distribution of said species. Although this species was typified by Drouet (1968), with the original collection by Bory set as a lectotype, it has accumulated so many disparities in reported habitat, morphology, and ecology that it will probably never, in our opinion, be confidently resolved. We, therefore, decided to name our morphologically similar species *M. pseudofavosus*, in reference to this unrevised expansive species. Although it cannot be said with certainty that no one will ever disambiguate *M. favosus* in the future, it is our opinion that this taxon, as well as numerous other unrevised taxa with conflicting records and unclear descriptions, would be better left to history.

*Microcoleus autumnalis*, as depicted by Strunecký et al. (2013), was morphologically very similar to *M. vaginatus* (sensu lato): cryptic and indistinguishable without the employment of molecular data (Komárek & Anagnostidis, 2005). The presence of an 11‐bp insert was previously used as an apomorphy to distinguish *M. vaginatus* from *M. autumnalis* (Boyer et al., 2002), but this marker was noted to be polyphyletic (Strunecký et al., 2013). To further complicate this issue, there are currently no available genomes of *M. autumnalis* in the NCBI database, and the confusion persists. Teneva et al. (2023) undertook a polyphasic characterization of *Phormidium autumnalis*, noting the transfer to *Microcoleus* was likely in error. However, this conclusion was weakly supported, as 16S rRNA gene phylogenies showed polyphyly, with strains of *P. autumnale* clustering in different branches of the phylogenetic tree. Furthermore, the clade that Teneva et al. (2023) considered “*Phormidium autumnale*,*”* was actually *Kamptonema animale* sensu Hindáková & Dvořák, 2023, and what Teneva et al. (2023) considered “*Kamptonema”* (the clade containing *P. etoshii* KR2008/49 and *P. animale* CCALA 140) is currently considered as *Laspinema* (sensu Dvořák et al., 2024). Thus, we continue to consider *M. autumnalis* as part of the genus *Microcoleus*. Although there are numerous 16S rRNA gene sequences labeled as “*P. autumnale”* (or *M. autumnalis*) in GenBank, they do not really simplify the taxonomic resolution, as they clustered into various branches in the phylogenetic tree (Figure S1). Most of the 16S rRNA gene sequences of *M. autumnalis* or *P. autumnale*, however, could be found in the larger branch of the phylogenetic tree comprised of clades *M. atroviridis* (M1), *M. tentaculus* (M2), *M. crotalus* (M3), *M. attenuatus* (M11), M14, M15, *M. occultus* (M16), and M21. Although cultivated strains from these clades within our collection bore some superficial similarity to *M. vaginatus* sensu stricto, none of them could be considered morphologically cryptic. A further complication was posed by the reported ecology of *M. autumnalis*: Most recordings of *M. autumnalis* came from freshwater habitats whereas most of the cultivated strains in our dataset originated from terrestrial habitats. Although efforts were made to align the putative species within our dataset to previously described, but unrevised species—where possible—rather than establishing new species, we lacked sufficient data to confidently delineate *M. autumnalis* sensu stricto. Potential matches included the putative species M14 and M15, which encompassed genomes of aquatic cyanobacteria. However, since these genomes were derived from metagenomic studies, we were unable to examine the morphology of these clades. Therefore, further investigation is required to definitively delineate *M. autumnalis* sensu stricto and determine its taxonomic position relative to its counterpart, *M. vaginatus*.

Another species that presented an interesting conundrum was M16 (*Microcoleus occultus*). This species formed a well‐supported, distinct clade among the *M. vaginatus* continuum, but was comprised solely of four herbarium‐derived genomes: two of *Phormidium subfuscum* (L.J. Von Heufler, 1864 and V.B. Wittrock, 1866), one *P. uncinatum* (H. L. D., 1896), and one *M. vaginatus* (F.B. Wartman, 1855). Despite the fact that these samples originated from varied geographic locations (United States of America, Italy, Germany, and Sweden) and our large‐scale sampling, we did not obtain any extant population of this species. Despite the lack of living strains, we erected this clade as a novel species, *M. occultus*, with the morphological description being based on microscopic observations of the herbarium items and on historical observations and descriptions from the original authors/collectors of the given herbarium samples. The lack of living strains in our dataset hints toward an even greater and still unexplored diversity within *Microcoleus*. We hope that a living population of this species will be discovered, whether by our own sampling efforts or by efforts of other teams focusing on the diversity and evolution of cyanobacteria. In any case, this is a taxonomic revision based on the herbarium‐derived genome.

Lastly, species M1 (*Microcoleus atroviridis*), M2 (*M. tentaculus*), M3 (*M. crotalus*), M4 (*M. toxifilus*), M8 (*M. pellucidapex*), and M9 (*M. malodorus*) all formed distinct, well‐supported clusters in the phylogenomic analysis, possessed distinct apomorphy or a combination of traits, and exhibited lower levels of gene flow between species than within species. Furthermore, we were unable to match these clades to any previously described and unrevised species of *Microcoleus*; thus, we erected these clades as novel species based on the genomic divergence, levels of gene flow, phylogenomic analysis, and morphological apomorphies.

Although we were able to resolve most of the putative species in our dataset, some outliers remained. The M13, M14, M15, M19, M20, and M21 “clades” consisted of either singletons or solely as metagenomes, without any available morphological or ecological data. Although they likely represent distinct species, we were unable to resolve them taxonomically. Also worth mentioning are the M17 (*Microcoleus anatoxicus*) and M18 clades. The M17 clade contained *M. anatoxicus* PTRS1, PTRS2, and PTRS3 (Conklin et al., 2020) and could be confidently regarded as such. Its sister clade, M18, however, contained a genome of *Tychonema bourellyi* (FEM GT703) published by Pinto et al. (2017). Although the taxonomic identity of the isolate remains uncertain, its close relationship to *M. anatoxicus* was further supported by the 16S rRNA gene phylogenetic tree (Figure S1). In this tree, most *Tychonema* sequences, including *T. bourellyi*, *T. bornetii*, and *T. tenue* SAG 4.82 (the only available sequence of the type species *T. tenue* sensu Anagnostidis & Komárek, 1988), formed a sister branch to *M. anatoxicus*, reinforcing their phylogenetic proximity. The keritomization of cell content is considered an apomorphy of *Tychonema*. However, this trait was observed in *Microcoleus* (Brown et al., 2025; Stancheva et al., 2024) and in the closely related *Kamptonema* (Hindáková & Dvořák, 2023) and is likely a response to low‐nitrogen environment rather than a stable apomorphy (Stancheva et al., 2024). It is becoming increasingly clear that *Tychonema* and *Microcoleus* are very closely related. However, resolving the taxonomy of this branch requires further detailed study and lies beyond the scope of this paper.

In the course of this study, we employed a framework proposed in Dvořák et al. (2025), integrating phylogenomic data, whole‐genome analysis, similarity values, genomic divergence, and population‐level morphological analysis. This framework offers a comprehensive view of species relationships, allowing for a much deeper understanding of genetic and phenotypic diversity. The combination of genomic and morphological data provides a more nuanced approach, bridging the gap between molecular genetics and observable traits, and ultimately leading to more robust species classifications. The use of herbarium‐derived genomes adds another critical layer to this framework, incorporating historical genetic data into the analysis. This not only improves the accuracy of modern taxonomic resolutions but also allows for the comparison of contemporary and historical specimens, potentially revealing shifts in species characteristics over time. By advancing this integrative framework, we aimed to establish a more precise and holistic understanding of Cyanobacterial taxonomy, with the ultimate goal of bringing taxonomic decisions in this important and ancient group of organisms as close to nature as possible.

In conclusion, we recognized 21 separate clades within the *Microcoleus vaginatus* group, representing 21 distinct species (Skoupý et al., 2024). Of these, three fit the description of previously described species or could be matched to existing species based on phylogenomic analysis: *M. vaginatus* sensu stricto, *M. attenuatus*, and *M. anatoxicus*. Furthermore, 11 clades could be delineated as novel species: *M. pseudofavosus*, *M. ladakhensis*, *M. drouetii*, *M. harperi*, *M. toxifilus*, *M. pellucidapex*, *M. malodorus*, *M. tentaculus*, *M. atroviridis*, *M. crotalus*, and *M. occultus*. Lastly, seven putative species remained uncharacterized due to a lack of sufficient data.

## AUTHOR CONTRIBUTIONS

**Svatopluk Skoupý:** Conceptualization (equal); data curation (equal); formal analysis (equal); investigation (lead); methodology (equal); visualization (lead); writing – original draft (lead); writing – review and editing (equal). **Aleksandar Stanojković:** Conceptualization (equal); data curation (equal); formal analysis (equal); investigation (equal); methodology (equal); software (equal); writing – original draft (equal); writing – review and editing (equal). **Jeffrey R. Johansen:** Conceptualization (equal); data curation (equal); formal analysis (equal); investigation (equal); methodology (equal); writing – original draft (equal); writing – review and editing (equal). **Dale A. Casamatta:** Conceptualization (equal); data curation (equal); formal analysis (equal); investigation (equal); methodology (equal); writing – original draft (equal); writing – review and editing (equal). **Callahan McGovern:** Conceptualization (equal); data curation (equal); writing – original draft (equal); writing – review and editing (equal). **Anne D. Jungblut:** Conceptualization (equal); data curation (equal); formal analysis (equal); methodology (equal); resources (equal); writing – original draft (equal); writing – review and editing (equal). **Jutta Fastner:** Data curation (equal); methodology (equal); resources (equal); writing – review and editing (equal). **Petr Dvořák:** Conceptualization (equal); data curation (equal); formal analysis (equal); funding acquisition (lead); investigation (equal); methodology (equal); project administration (lead); resources (lead); supervision (lead); writing – original draft (equal); writing – review and editing (equal).

## Supporting information


**Figure S1.** Phylogenetic reconstruction of 967 16S rRNA sequences of Microcoleus sp.

**Figure S2.** Whole‐genome phylogeny of *Microcoleus vaginatus* speciation continuum. This figure relates to Figure 7 of the main text.

**Table S1.** Concentration of anatoxin‐a, dihydroanatoxin‐a, and homoanatoxin‐a in selected strains measured using LC–MS/MS.
