# Supplementary material for: Population and herbarium genomics provide a comprehensive framework for a revision of Microcoleus (Cyanobacteria)
Source: J Phycol. 2026 Mar 18;62(2):454–76. doi: 10.1111/jpy.70145 (PMC13103696; doi:10.1111/jpy.70145)
Supplement: Supplementary file 2 — Figure S2. Whole‐genome phylogeny of Microcoleus vaginatus speciation continuum. This figure relates to Figure 7 of the main text. [file JPY-62-454-s003.pdf]

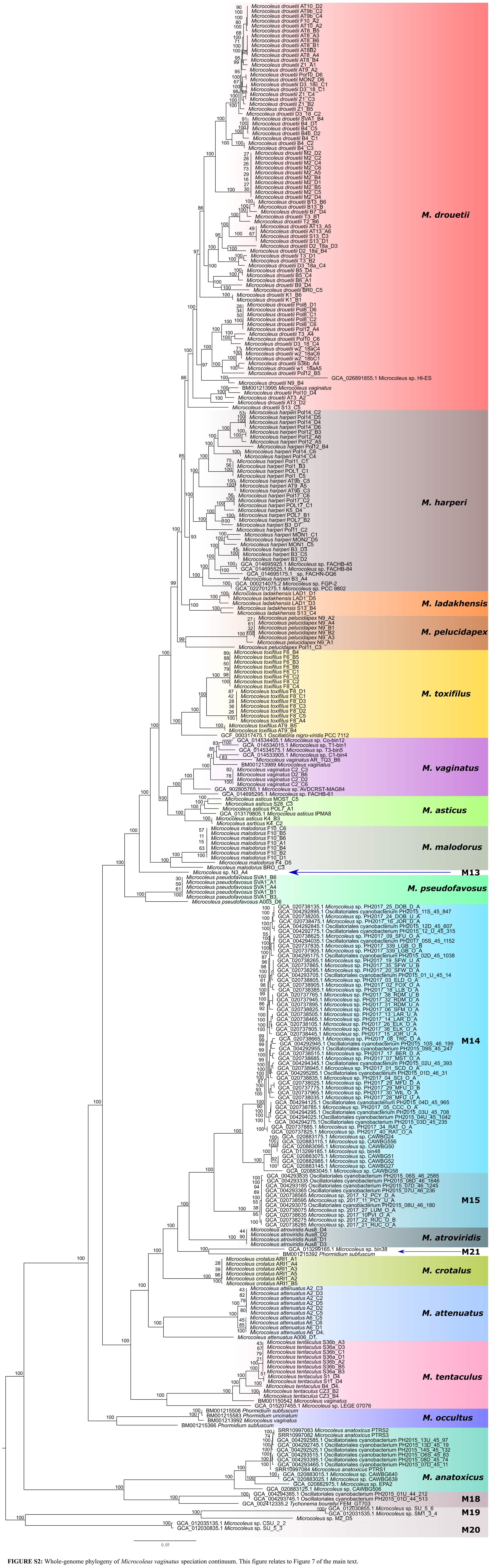

FIGURE S2: Whole-genome phylogeny of *Microcoleus vaginatus* speciation continuum. This figure relates to Figure 7 of the main text.
