# Supplementary material for: Population and herbarium genomics provide a comprehensive framework for a revision of Microcoleus (Cyanobacteria)
Source: J Phycol. 2026 Mar 18;62(2):454–76. doi: 10.1111/jpy.70145 (PMC13103696; doi:10.1111/jpy.70145)
Supplement: Supplementary file 3 — Table S1. Concentration of anatoxin‐a, dihydroanatoxin‐a, and homoanatoxin‐a in selected strains measured using LC–MS/MS. [file JPY-62-454-s002.docx]

| **Strain** | **Anatoxin-a [µg ∙ g^−1^ DW]** | **Dihydroanatoxin-a [µg ∙ g^−1^ DW]** | **Homoanatoxin-a [µg ∙ g^−1^ DW]** |
| --- | --- | --- | --- |
| *Microcoleus. toxifilus* F8_D3 | 0.64 | 0.10 | 31.16 |
| *M. toxifilus* F8_D1 | 2.79 | 0.15 | 49.93 |
| *M. toxifilus* F8_C3 | 8.19 | 0.08 | 60.83 |
| *M. toxifilus* F8_C1 | 2.02 | 0.34 | 70.83 |

**Table S1:** Concentration of anatoxin-a, dihydroanatoxin-a and homoanatoxin-a in selected strains measured using LC-MS/MS. DW, dry weight
