# Supplementary material for: Population and herbarium genomics provide a comprehensive framework for a revision of Microcoleus (Cyanobacteria)
Source: J Phycol. 2026 Mar 18;62(2):454–76. doi: 10.1111/jpy.70145 (PMC13103696; doi:10.1111/jpy.70145)
Supplement: Supplementary file 1 — Figure S1. Phylogenetic reconstruction of 967 16S rRNA sequences of Microcoleus sp. [file JPY-62-454-s001.pdf]

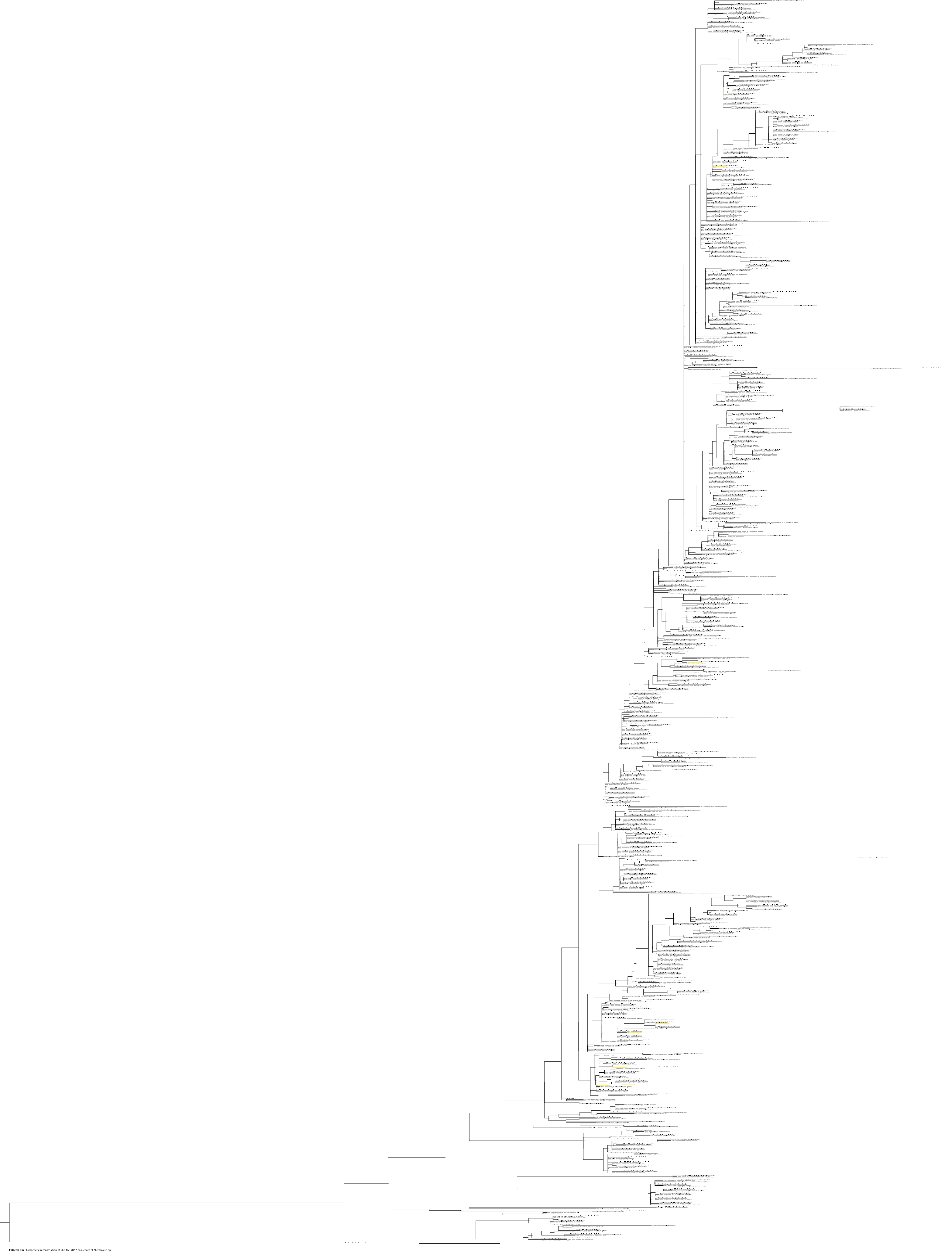

FIGURE S1: Phylogenetic reconstruction of 967 16S rRNA sequences of Microcoleus sp.
